# Potential Endocrine Disruption of Cyanobacterial Toxins, Microcystins and Cylindrospermopsin: A Review

**DOI:** 10.3390/toxins14120882

**Published:** 2022-12-17

**Authors:** Antonio Casas-Rodriguez, Ana M. Cameán, Angeles Jos

**Affiliations:** Faculty of Pharmacy, University of Seville, 41004 Seville, Spain

**Keywords:** microcystins, cylindrospermopsin, endocrine disruption, steroidogenesis, thyroid hormones

## Abstract

Microcystins (MCs) and cylindrospermopsin (CYN), although classified as hepatotoxins and cytotoxins, respectively, have been shown to also induce toxic effects in many other systems and organs. Among them, their potential endocrine disruption (ED) activity has been scarcely investigated. Considering the increasing relevance of ED on humans, mammals, and aquatic organisms, this work aimed to review the state-of-the-art regarding the toxic effects of MCs and CYN at this level. It has been evidenced that MCs have been more extensively investigated than CYN. Reported results are contradictory, with the presence or absence of effects, but experimental conditions also vary to a great extent. In general, both toxins have shown ED activity mediated by very different mechanisms, such as estrogenic responses via a binding estrogen receptor (ER), pathological changes in several organs and cells (testis, ovarian cells), and a decreased gonad-somatic index. Moreover, toxic effects mediated by reactive oxygen species (ROS), changes in transcriptional responses on several endocrine axes and steroidogenesis-related genes, and changes in hormone levels have also been reported. Further research is required in a risk assessment frame because official protocols for assessment of endocrine disrupters have not been used. Moreover, the use of advanced techniques would aid in deciphering cyanotoxins dose-response relationships in relation to their ED potential.

## 1. Introduction

The endocrine system is a collection of numerous glands that directly secrete different hormones into blood circulation and includes different axes, such as hypothalamic-pituitary-adrenal/interrenal (for fish) (HPA/HPI), hypothalamic-pituitary-gonad (HPG), and hypothalamic-pituitary-thyroid (HPT) [1]. Each axis regulates different functions of the body, including metabolism, growth, and development, via axis-specific hormones. The HPA axis mainly controls bodily responses to stress by glucocorticoids, cortisol, or corticosterone, while the HPG axis coordinates reproduction by steroid hormones, synthesized from cholesterol in a process called steroidogenesis, and the HPT axis regulates energy metabolism and development by thyroid hormones [2,3].

This system can be altered by different substances called endocrine disruptors (EDs) [4]. In the literature, other authors have defined ED as an exogenous substance that interferes with the function of hormonal systems and produces a range of developmental, reproductive, neurological, immune, or metabolic diseases in humans and wildlife [5]. According to the World Health Organization (WHO), an ED is “an exogenous substance or mixture that alters function(s) of the endocrine system and consequently causes adverse health effects in an intact organism, or its progeny, or (sub)population” [6]. Moreover, for the European Food Safety Authority (EFSA), an ED is defined by three criteria: (i) the presence of an adverse effect in an intact organism or (sub)population; (ii) the presence of an endocrine activity; and (iii) a plausible or demonstrated causal relationship between the endocrine activity and the adverse effect [7]. During the last few years, there has been an increasing public concern for human exposure to EDs through water and food consumption, and, due to their widespread detrimental effects, the WHO has included EDs among high-priority research fields since 2010 [8]. Furthermore, epidemiological data show increases in incidence and prevalence of diseases associated with endocrine-disrupting chemicals, such as breast, prostate and testis cancer, diabetes, obesity, and decreased fertility over the last 50 years [9,10].

Regarding their mechanism of action, EDs can disrupt the endocrine system in a wide variety of ways, such as activating or inactivating endocrine target receptors, interfering with the synthesis, metabolism, and transport of different hormones, or antagonizing their action [5,11]. Most known targets for EDs are nuclear and extranuclear receptors, such as estrogen receptors (ER), androgen receptors (AR), thyroid receptors (TR), or glucocorticoid receptors (GR), among others [12]. The binding of EDs to both ER or AR can activate or inhibit transcriptional or post-transcriptional mechanisms, with adverse effects on reproduction [13]. EDs can also inhibit synthesis or interfere with the metabolism of sex steroids by the disruption of normal action carried out by estrogens and androgens [14].

Due to the complexity of these toxic mechanisms, the EFSA Scientific Committee has published a scientific opinion about the evaluation of endocrine disruptor hazards, the scientific criteria for their classification, and the appropriate methods by which to evaluate them [7]. The EFSA includes the methods covered by the Organization for Economic Co-operation and Development (OECD) Conceptual Framework (CF) for Testing and Assessment of Endocrine Disrupters [15] in five different levels. These encompass Level 1 (existing information and non-test information), Level 2 (selected in vitro endocrine mechanistic/mode of action test methods), in vivo selective endocrine mechanistic screening methods in Level 3, in vivo apical tests (for adverse effects) including endocrine relevant endpoints in Level 4, and other comprehensive data concerning the more extensive parts of the life cycle in Level 5. Thus, a single assay cannot provide all the information needed to classify a substance as an ED, and various standardized assays are needed. Among these assays, those that detect substances that interact with estrogens, androgens, and thyroid hormones, or affect steroidogenesis, are of relevance. This research can be carried out using in vitro and in vivo assays included in Levels 2 and 3 of the abovementioned OECD CF. The Guidance Document on Standardized Test Guidelines for Evaluating Chemicals for Endocrine Disruption (GD 150) was originally published in 2012, and the last edition (2018) included new and revised OECD internationally harmonized test guidelines, assays validated at international level, and assays that are currently in the OECD validation process [16].

A wide variety of substances have been noted as EDs, including pesticides, industrial by-products, environmental pollutants, plastics, metals, food contaminants, and personal care products [17]. Furthermore, cyanobacterial blooms could be a source of diverse EDs, as it has been demonstrated in eutrophic waters in China (Lake Taihu), using in vitro reporter gene bioassays and chemical methods of determination like UPLC-MS/MS [18]. In this sense, cyanotoxins have been proposed as potential EDs due to their estrogenic potency and capacity of interference in the signaling of intracellular receptors that are important for hormonal regulation, reproduction, and the development of vertebrates [19]. 

Cyanobacteria are prokaryote photosynthetic organisms that can grow and form cyanobacterial blooms due to eutrophication, climate change, and anthropogenic activities. These cyanobacterial blooms are recognized worldwide as an environmental threat because they are capable of producing a wide range of secondary metabolites and bioactive compounds, with many considered to be toxins (cyanotoxins) [20,21]. Cyanotoxins can reach humans through diverse pathways, mainly orally through the consumption of contaminated water and food, although inhalation and dermal exposure during recreational activities are also common [22]. Exposure to cyanotoxins can lead to high toxic risks, so the WHO and the EFSA have catalogued cyanobacteria as an important health issue [23]. There are several types of cyanotoxins according to their target organ. The main toxins groups are hepatotoxins, cytotoxins, neurotoxins, dermatoxins, and irritant toxins [24]. Among cyanotoxins, microcystins (MCs) are the most studied and known because of their prevalence and toxicity, but cylindrospermopsin (CYN) is becoming increasingly relevant worldwide due to its wide distribution, bioaccumulation capacity, and toxic effects. 

The general structure of MCs is: cyclo(-D-Ala1-L-X2-D-erythro-β-methylAsp(iso-linkage)3-L-Z4-Adda5-DGlu(iso-linkage)6-N-methyldehydro-Ala7). The L-amino acid residues 2 (X) and 4 (Z) contribute to the major varieties of MCs, and more than 279 structural MCs variants have been reported [25]. Microcystin-LR (MC-LR) is the most frequently assessed variant, and is the most toxic in different models [26,27]. Other variants (MC-RR, -YR, -LF, -LA, -LY) have gained more interest due to their toxicity [28,29]. MCs are hepatotoxins and tumor promoters due to their strong potent inhibition of protein phosphatases 1 and 2A (PP1 and PP2A), induction of oxidative stress [30], and effects on cell signaling pathways [31]. Although the liver is the main target organ of MCs, other effects have been reported, such as neurotoxicity [32], reproductive toxicity [33], and endocrine disruption [3]. MCs cause reproductive toxicity via apoptosis, autophagy, cytoskeletal destruction, reproductive tumors, and endocrine disruption [33,34,35]. On the other hand, CYN is an alkaloid consisting of a tricyclic guanidine moiety combined with hydroxymethyluracil, which can be produced by *Cylindrospermopsis raciborskii* and *Chrysosporum ovalisporum* [36]. CYN appears to be a molecule with a wide range of toxic effects. The toxin primarily targets the liver, but it is also a general cytotoxin that attacks the eye, spleen, kidney, lungs, thymus, heart, etc. [37]. Concerning the mechanisms of action, CYN is well known by its inhibition of protein and glutathione synthesis, induction of oxidative stress, and cytochrome P450 seems to mediate its toxicity [24]. Its pro-genotoxic activity has also been studied [38] and its genotoxicity has been demonstrated in vitro and in vivo [39,40]. 

Regarding endocrine disruption by cyanotoxins, many studies have also demonstrated that MCs can disrupt the synthesis of steroid hormones, resulting in dysfunction of the endocrine system and reproductive toxicity, especially MC-LR [41]. Oziol and Boüaicha [42] were the first to show the in vitro estrogenic activity of MC-LR on ER, but they suggested that maybe other bioactive compounds present in the biomass could be the responsible for these effects, and not the cyanotoxin itself. In that sense, the extracts from complex cyanobacterial biomasses exhibited greater estrogenic potency and interference with androgen signaling, as well as greater cytotoxicity potency, than extracts from a single cyanobacterial species [43]. Furthermore, in zebrafish, the upregulation of vitellogenin (VTG) (a protein involved in oocyte maturation) is one of the most frequently used biomarkers for exposure to estrogenic compounds [44]. In that sense, Rogers et al. [19] found that *Microcystis* exposure produced a significant upregulation of this protein, but there were no significant effects after exposure to standard MC-LR.

In vivo, MC-LR affects the serum levels of hormones and their expression by affecting the HPG [45,46]. Through this axis, gonadotropin-releasing hormones (GnRHs) released from the hypothalamus stimulate the secretion of gonadotropin hormones (GtHs), including follicle-stimulating hormones (FSH) and luteinizing hormones (LH), by the pituitary. GtHs are then transported to the gonads to induce steroidogenesis and produce sex steroid hormones, such as 17β-estradiol (E2) and testosterone (T). Levels of these hormones, which modulate the reproductive process, can be altered by MC-LR [41,47]. In addition, several studies have shown that MC-LR can alter steroidogenesis by causing changes in the expression of genes involved in this process [3,48]. 

In the case of CYN, studies focusing on the potential endocrine disrupting effects of the toxin are very scarce. Some estrogenic effects have been detected in extracts of cyanobacteria containing CYN [49], but information of the estrogenic potency of CYN is very limited [50].

Considering all of these facts, the aim of this work is to compile the scientific literature existent since 2010 regarding this important topic, the potential estrogenic/antiestrogenic or androgenic/antiandrogenic effects, as well as the effects on steroidogenesis, and, finally, changes in thyroid hormones induced by MCs and CYN.

## 2. Microcystins

### 2.1. Estrogenic/Antiestrogenic Effects of MCs and Consequences on Reproduction Toxicity

The most important features derived from the in vitro and in vivo studies performed with MCs, especially focused on the potential estrogenic disruption effects of MC-LR, compiled after revision of the scientific literature, are shown in Table 1.

Several in vitro and in vivo methods have been used to evaluate the estrogenic activity of xenoestrogens. Among the in vitro methods, the following assays can be included: the competitive estrogen receptor (ERs) binding assay, the proliferation assay (E-screen) in the human breast cell line MCF-t7, the vitellogenin (VTG) induction assay in primary hepatocytes from fish, and the receptor gene assays using transgenic human cell lines or yeasts [42]. 

The first study to determine the estrogenic and anti-estrogenic capacities of MC-LR and Nodularine–R (NOD-R) in vitro was carried out in a stably transfected cell line (MELN) with an estrogen-regulated luciferase gene [42]. The activation of the luciferase gene indicated that both toxins, MC-LR and NOD-R, at low concentrations, presented estrogenic potential; higher with MC-LR than with NOD-R, likely due to the indirect interaction with ERs. This weak estrogenic activity was inhibited when a pure estrogen receptor antagonist was added to the cells. At high MC-LR concentrations (40.2–60.4 nM), decreased luciferase activity was reported, and this fact can be explained by the cytotoxic effect observed using the 3-(4,5-dimethyl-2-thiazolyl)-2,5-diphenyl-2H-tetrazolium bromide (MTT) assay. No synergistic estrogenic effects were observed with the mixture of MC-LR and NOD-R. The authors suggested that the estrogenic properties of these cyanotoxins could be mediated by signaling phosphorylation pathways (similarly to okadaic acid). On the other hand, oxidative stress could be mediated by the formation of reactive oxygen species (ROS), which cause modulation of the oxidized/reduced ratio of signaling proteins. 

In addition to this study, it is important to highlight that only a few studies have determined the potential endocrine disruption activity of cyanobacterial cells or pure cyanotoxins through interactions with the signaling pathways of the estrogen receptor (ER), androgen receptor (AR), glucocorticoid receptor (GR), and retinoid receptor (RAR) [43,49,51,52]. None of them were performed following the requirements of the OECD guidelines [53,54,55]. Stepánková et al. [43] demonstrated that extracts from complex cyanobacterial biomasses exhibited greater estrogenic potency and interference with androgen signaling, as well as greater cytotoxicity potency, than extracts of single cyanobacterial species. Similarly, Sychrová et al. [49] investigated the estrogenic potency of aqueous extracts and exudates of seven species of cyanobacteria and two algal species by using in vitro trans-activation assays. Most exudates exhibit estrogenic activities, with the greatest estrogenic potency shown by exudates from *Microcystis aeruginosa*. Moreover, *Scenedesmus quadricauda* exudates and extracts from *Aphanizomenon flos-aquae* were antagonistic to the ER when they were coexposed to 17B-estradiol. In agreement with previous work, estrogenic potency revealed the role of compounds not specific to cyanobacteria, but present in algae, probably phytoestrogens.

In the same way, Jonas et al. [51] investigated the in vitro estrogenic, anti-androgenic, glucocorticoid, and retinoid-like activities of three species of cyanobacteria: *M. aeruginosa, Planktothrix agardhii*, and *Aphanizomenon gracile*. They confirmed production by phytoplankton species of estrogenic and retinoid-like compounds. The endocrine activities detected might not be directly reflected by whole organism assays. In parallel, the authors carried out in vivo experiments using transgenic zebrafish embryos, and the estrogenic effects were not detected because they were masked by high toxicity. Corresponding to the retinoid-like activity of *A. gracile* extracts, teratogenic effects were detected, and an increased body length of embryos was also observed after exposure to biomass extracts. All of these findings highlight the importance of investigating the activity of estrogens, androgens, retinoids, and other bioactive compounds present in cyanobacterial blooms. In contrast, more recently, Mallia et al. [52] investigated the in vitro endocrine activities of 26 cyanobacterial cultures of *Microcystis* or *Planktothrix* species by screening them in estrogen-, androgen-, and glucocorticoid-responsive reporter gene assay (RGA), and the results were not conclusive.

Zebrafish are one of the experimental models more frequently employed to study the potential estrogenic activities of cyanotoxins, and also, in some cases, the reproduction of fish [19,41,44,47,48,51,56,57,58,59,60,61,62,63,64]. Only one study has demonstrated pronounced estrogenic effects of *M. aeruginosa* exudates on the reproductive, physiological, and molecular characteristics of *Daphnia magna* (zooplankton) [65]. A few studies have been carried out on mammals, and some of them have explored the potential consequences on the reproductive toxicity of MCs [3,66,67,68], although the number of studies is growing [3]. 

In zebrafish, one of the first studies suggested that VTG abundance increased in MC-LR-exposed zebrafish brains, and this was explained because the toxin might mimic the effects of endocrine-disrupting chemicals (EDCs) [56]. In zebrafish larvae exposed to purified MC-LR or lyophilized *M. aeruginosa* cells containing MC-LR, an upregulation of VTG genes was observed in the case of exposed *Microcystis* larvae, although not in the group exposed to MC-LR [19]. VTG are a group of lipoproteins produced in the liver in response to estrogens, and are transported through the blood and deposited in the developing oocytes of female fish [69]. The fact that only *Microcystis* cells showed induction of VTG could be explained by the presence of substances produced by these cells, such as phytoestrogens. 

Because of the endocrine disorders induced by MC-LR, the toxin exhibits reproductive toxicity, as has been reviewed [33,34,35], and zebrafish have been chosen as experimental models to understand the effects of MCs on fish. Male and female zebrafish exposed by subchronic immersion (30 days (d)) to MC-LR suffered from toxic effects in terms of gonads, hatchability, and hormone levels; whole body VTG levels significantly increased in females, while they decreased in males [57]. Moreover, the VTG1 transcriptional level was significantly reduced in the livers of both sexes. Marked histological lesions were observed in the livers, ovaries, and testes of treated fish, and a significantly elevated apoptosis rate was observed in ovaries instead of testes. These results confirmed that female zebrafish were more vulnerable than males, and that MC-LR did not exert any estrogenic effects on adult zebrafish [57].

The sex-dependence effects of MC-LR exposure on fish reproduction was extensively investigated by Liu et al. [41], and some important molecular biomarkers related to gametogenesis in zebrafish were altered after MC-LR exposure (1–20 µg/L for 30 d). Thus, levels of E2, T and 11-keto testosterone (11-KT), and FSH were increased in serum from all of the treated females, while T, FSH, and LH levels changed in all of the treated males. Histopathological changes were observed, with retarded oogenesis and spermatogenesis. Transcriptional changes in 22 genes on the HPG axis exhibited sex-specific responses. 

Zhao et al. [58] investigated the effects of MC-LR on zebrafish reproduction by affecting oogenesis, as well as the effects on sex hormones and the transcription of genes on the HPG axis. Decreased egg production after exposure to MC-LR (>10 µg/L) and increased concentration of E2 and VTG at 10 µg/L MC-LR was reported, whereas at higher concentrations of 50 µg MC-LR/L, concentrations of E2, VTG, and T declined. Moreover, changes in the transcription of steroidogenic pathway genes, and in numerous intra- and extra-ovarian factors, which regulate oogenesis, were detected. Female zebrafish exposed to MC-LR experienced decreased egg production, fertilization, and hatching rates, which may reflect a defect in folliculogenesis and possible poor oocyte quality. Consequently, the changes in these endpoints could affect unexposed F1 or F2 generations, leading to potential multigenerational and transgenerational effects, respectively. Later, Hou et al. [44] studied the effects of MC-LR during life cycle exposure in zebrafish and hatchlings (5 d post fertilization), and demonstrated for the first time that MC-LR induced growth inhibition and decreased ovary weight and ovarian ultra-pathological lesions. Decreased ovarian T levels indicated that MC-LR disrupted sex steroid hormone balance. Moreover, the significantly upregulated transcription of brain FSHβ and LHβ along the ovarian ERα, FSHR, and LHR suggested that positive feedback regulation in the hypothalamic-pituitary-gonadal-liver (HPGL) axis was induced as a compensatory mechanism for MC-LR damage [44]. In contrast, ovarian VTG content and hepatic ERα and VGT1 expression were downregulated, which agreed with reduced vitellus storage found in the histopathological study. In summary, MC-LR impairs the development and reproduction of female zebrafish by disrupting the transcription of related HLPG-axis genes. 

The same authors [59] evidenced that MC-LR caused gonadal development retardation through disrupting the growth hormone/insulin-like growth factors (GH/IGFs) system, which plays an important role in the endocrine regulation of fish growth. Thus, zebrafish hatchlings (5 d post fertilization) exposed to MC-LR for 90 days until sexual maturity showed delayed ovarian maturation and sperm development, as well as lesions in the brain and liver. The retarded gonadal development was parallel to an inhibition of the GH/IGFs system, which was characterized by significant decreases in some mRNA expression profiles of the genes in the brain, hepatic, and gonadal levels in males [59]. In this work, the gonadal development of males was more vulnerable than that of females after MC-LR exposure. 

Nevertheless, Quiao et al. [57] reported that adult zebrafish females exhibited more susceptibility than males, and Liu et al. [41] showed that exposure to MC-LR could disrupt the function of the HPG endocrine system and indicated sex-dependent effects of MC-LR on fish reproduction. These discrepancies indicate that more studies are needed to investigate if the sex-dependent effects of MC-LR could depend on the tested life stage, or on the general conditions assayed. 

Su et al. [60] also demonstrated that life cycle exposure to MC-LR caused endocrine disruption in male zebrafish, with growth inhibition and organic and functional damage of the testis. A significant decrease in the T/E2 ratio, a sensitive biomarker of abnormal sex hormone levels in fish [70], indicated that MC-LR disrupted sex steroid hormones balance. The changes in transcriptional responses of HPG-axis-related genes revealed that the toxin promoted the conversion of T to E2 in circulating blood, and the upregulation of vgt1 mRNA expression in the liver indicated that MC-LR-induced estrogenic-like effects [60].

More recently, a study carried out on female zebrafish subchronically exposed to MC-LR (30 d) investigated the reproductive toxicity of the toxin by affecting oocyte development and fertilized eggs [64]. Pathological changes were observed in the ovaries after MC-LR exposure, and a significant increase in the rate of the zebrafish oocytes germinal vesicle breakdown (GVBD), malformations of the offspring, and decreased cAMP and VTG levels were detected at the highest concentration assayed. Moreover, the phosphorylation levels of the extracellular signal-regulated kinases (ERK) were elevated in the ovaries, as well as phosphorylated cyclin B levels. The authors suggested that MC-LR promotes oocyte maturation by activating the ERK1/2 (the most extensively studied members of the ERK family) and maturation-promoting factor (MPF) signaling pathways, and that cAMP is involved in this process. 

In addition to the studies that demonstrated the endocrine-disrupting activity of MC-LR pure standard in zebrafish, the effects of *M. aeruginosa* on the HPGL axis in female zebrafish after short-term exposure (96 h) were investigated by Liu et al. [62]. Not only did the cyanobacteria cause histological lesions in the liver and gonads, but the fertilization rate and hatchability of eggs spawned in treated groups were also decreased, and transgenerational effects could appear. Moreover, *M. aeruginosa* decreased E2 and T plasma levels, and the vtg1 transcriptional level was decreased in the liver, whereas plasma VTG protein levels increased. The downregulation of the hepatic *vtg1* gene could result from decreased E2 levels in the plasma, and from liver lesions caused by *M. aeruginosa*. In contrast, plasma VTG levels increased in a concentration-dependent manner, and this fact could be caused by the impairment of the ovaries, making it difficult for plasma VTG to incorporate into the oocytes. Similarly, Quiao et al. [57] found that MC-LR decreased hepatic *vtg1* gene expression and increased VTG levels in zebrafish, as previously mentioned.

In the same model, synergism after combined exposure of MC-LR and nitrite causing reproductive dysfunction by interferences with the HPGL axis was also demonstrated in male zebrafish [61]. Both MC-LR and nitrite caused concentration-dependent effects, including testicular pathological changes, but the effects were more consistent in comparison to the single exposure groups. Exposure to MC-LR or nitrite alone significantly decreased T levels by the downregulation of gene expression in the HPLG axis, and interaction between them was significant. In contrast, E2 levels and the transcriptional levels of *cyp19a1b*, *cyp19a1a*, and *vtg1* were increased with MC-LR concentrations, confirming the estrogen-like effects of MC-LR, as previously reported [60]. 

Moreover, the recovery mechanism of the reproductive function of adult zebrafish exposed to MC-LR exposure (0–50 µg/L for 21 d) and later transferred to MC free water for another 21 d was demonstrated by Kawan et al. [47]. In this work, after MC-LR exposure, several changes were reported: histopathological lesions in the gonads; a decreased percentage of mature oocytes and number of spawned eggs; decreased fertilization and hatching rates; increased concentrations of E2, T, and VTG in females; some gene transcriptions of the HPG axis were changed; and ovarian protein levels were increased. However, after 21 d of depuration (in water MCs free) the reproductive changes were also reversible. 

In mammals, MC-LR induced female reproductive toxicity in mice after 28 d of exposure (i.p.), with reduced relative ovary weight and pathological changes in ovaries. MC-LR induced decreases in progesterone (P4), but no FSH or LH effects were reported. The alterations of the estrus cycle could be explained as a result of direct impact on the ovaries rather than indirect actions from the hypothalamus or pituitary. The toxin was detected in the ovaries of treated mice [66]. The same authors investigated the effects of MC-LR in mice after oral exposure (3 or 6 months) and the toxin stimulated follicle atresia, decreased developmental follicles, reduced the gonadosomatic index (GSI), and confirmed the changes in estrus cycles [67]. 

Finally, an extensive study was performed in rats, providing data from which to understand the endocrine-disrupting effects of MC-LR [3]. In this interesting and novel work, after a single i.p. injection of MC-LR (median lethal dose), the histopathology of several organs (hypothalamus, pituitary, adrenal, ovary, and thyroid) was analyzed, and concentrations of hormones in serum and gene expression of the HPA, HPG, and HPT axes were examined. The authors suggested that MC-LR affected the three axes. The changes in concentrations of hormones were not dose-dependent, and they hypothesized that the threshold for endocrine-disrupting effects of MC-LR might be less than 36.5 µg/kg b.w. In addition, they reported that non-monotonic dose responses (NMDRs) of hormones and genes were observed. NMDRs are relatively common in studies of endocrine-disrupting chemicals (EDCs), and it would be suitable to investigate endocrine disorders induced by smaller concentrations of MC-LR. Further studies are needed to confirm the effects of MC-LR and other congeners on the three axes. 

In conclusion, the estrogenic effects of MC-LR have been demonstrated in different models (mainly in zebrafish), and several experiments have demonstrated that the toxin is able to disrupt the transcription of related HPGL axes through several mechanisms. In mammals, the toxin affected concentrations of hormones in the serum and gene expressions of the HPA, HPG and HPT axes, and the threshold level for endocrine-disrupting effects of MC-LR proposed in rats still needs to be confirmed. More studies are needed in several directions: (1) to elucidate if MC-LR effects are sex-dependent; (2) to investigate the main action of pure MC-LR in comparison to other substances present in cyanobacterial blooms (which may be phytoestrogens); (3) in vitro (level 2) and in vivo studies (level 3) following OECD guidelines with several pure MCs congeners (MC-LR, MC-RR, MC-YR) are needed in order to know if they are able to interact with ER, e.g., their effects in uterine weight or uterotrophic response in mammals have not been investigated yet; 4) further studies to explore the combination of cyanotoxins and other pollutants (metals, pesticides, plastics), some of them considered as EDs.

**Table 1:** Estrogenic/antiestrogenic effects of MCs and consequences on reproduction toxicity.

Toxin	Experimental Model	Assays Performed	Concentration Ranges and Exposure Conditions	Main Results	References
Pure MC-LR standards	Zebrafish	Determination of protein profile in the brain using a 2-DE gel by silver staining. Identification of altered proteins by mass spectrometry.	2 and 20 µg/L for 30 days (d)	MC-LR induced the expression of VTG3 and increased the abundance of VTG 1 in brains.	[56]
Pure MC-LR standard and *Microcystis* from lyophilized cells of *Microcystis aeruginosa*	Larval zebrafish	Microarray analysis. Determination in whole larvae of *vtg1* expression by qRT-PCR.	0–1000 µg/L MC-LR for 96 h 50 mg of lyophilized cells/L	Around 1000 genes with significant changes in expression were found after MC-LR exposure; 69 of these genes were differentially exposed in both 100 and 1000 µg/L MC-LR. These genes were related to detoxification and metabolism, cell signaling and development, or liver function. Around 371 genes were differentially expressed in *Microcystis* treatment. Only *Microcystis* caused an upregulation of *vtg1* expression.	[19]
MC-LR standard	Male and female Zebrafish	Histology of liver and gonads. Determination of E2 and T gonads levels by ELISA. Expression of *vtg1* mRNA in liver by RT-PCR. Apoptotic rate of gonads by flow cytometry. Expression of apoptosis-related genes in gonads by RT-PCR. Determination of gonadal caspase-3 by colorimetric assay kit.	1, 5 and 20 µg/L for 30 d	E2 concentration in females significantly decreased, and E2 levels in males decreased. No significant changes in T levels in either males and in females. Significant elevated apoptosis rate in ovaries, instead of testes. The downregulation of *Bcl-2* (anti-apoptotic gene) expression in both females and males confirmed MC-induced apoptosis through the mitochondrial pathway in reproductive systems of fish. In treated female zebrafish, caspase-3 was significantly elevated, although its transcriptional levels decreased.	[57]
Cyanobacterial biomass extracts (MC-LR; -RR; -YR)	Zebrafish embryos	Teratogenicity was examined. Neuroactivity or toxicity with a locomotor assay. Evaluation of estrogenic activity.	0.3, 1, 3 and 10 g dw/L for 120 hpf	Teratogenic effects in zebrafish embryos exposed to extract of *A. gracile* at 1 g dw/L. Deformities of the tip of the tail and spine. Edema of the heart and trunk, small head and yolk retention, at high concentrations. Estrogenic effects were not significant. Significant differences in locomotion compared to control for 0.3 g dw/L extracts of *M. aeruginosa* and *P. agardhii*.	[51]
	MVLN cell line transfected, MDA-kb2 cells transfected, P19/A15 cell line transfected	Determination of estrogenic, (anti-androgenic), glucocorticoid, and retinoid-like activities using luciferase substrate.	0.03125–2 g dw/L for 24 h	The assay revealed concentration-dependent retinoid-receptor-mediated activity in the biomass extracts of all tested cyanobacteria. Estrogenic potency was detected in all samples. No androgenic, glucocorticoid, or antiandrogenic activities were detected in any of the tested biomass extracts. A potential estrogenic activity was indicated for the extracts of *A. gracile*, *P. agardhii*, and *M. aeruginosa* at 0.3 g dw/L.	
MC-LR standard	Male and female Zebrafish	Histological examination of ovaries. Determination of E2, T, and VTG plasma levels by ELISA. Expression of different genes (*gnrh*, *lh*, *fsh*) in brain, liver, and ovary by RT-PCR.	2, 10 and 50 µg/L for 21 d	10 µg/L MC-LR caused stronger effect on increasing sex hormones and related regulating genes and inducing maturation, whereas 50 µg/L MC-LR induced a greater impact on the inhibition of maturation and ovulation in fish. MC-LR strongly impaired follicular development by influencing intra- and extra-ovarian factors involved in these processes.	[58]
MC-LR standard	Male and female Zebrafish	Determination of E2, T and VTG levels by ELISA. Expression in brain, ovary, and liver of *gnrh*, *lh*, *fsh*, and *vtg* genes by RT-PCR.	0.3, 3 and 30 µg/L for 90 d	Significantly decreased ovarian T levels. Expression of *fsh* and *lh* genes increased. Significant downregulation was detected in *vtg1* mRNA expression in the liver of female zebrafish. *vtg* level was not affected in the 0.3 mg/L MC-LR group, but was significantly downregulated in the 3 and 30 mg/L groups.	[44]
MC-LR standard	Male and female Zebrafish	Histopathological observation of gonads. Analysis of oogenesis and spermatogenesis. Determination of E2, T, FSH, LH, and 11-KT plasma levels. Expression in brain and gonads of 22 genes implicated in HPG axis by RT-PCR. Determination of *17βhsd* and *CYP19a* protein expression in gonads by Western blot.	1, 5 and 20 µg/L for 30 d	Loss of contact between follicular cells and oocytes in ovary and cellular deterioration in testes. Decrease in both vitellogenic and post-vitellogenic oocytes and in the number of spermatozoa. In all fish serum levels of E2, T, 11-KT, and E2/11-KT ratio increased. E2/T ratio decreased in females. Both serum levels of FSH and LH increased in females and decreased in males. In females, mRNA levels of *cyp19b*, *erβ*, and *fshr* increased, while *gnrh2* expression decreased. In males, significant downregulation of *gnrh2*, *erβ*, and *fshr*, and significant up-regulation of *17βhsd*. In both males and females, protein levels of *17βhsd* and *CYP19a* increased.	[41]
MC-LR standard	Male Zebrafish	Histopathological analysis of testis. Determination of E2 and T testis levels by ELISA. Expression of different genes (*gnrh*, *lh*, *fsh*) in brain, liver, and testis by RT-PCR.	0.3, 3 and 30 µg/L for 90 d	MC-LR exposure could exert chronic toxicity, including growth inhibition, testicular damage, and sperm maturation delay, as well as the imbalanced secretion of sex hormones (increased secretion of T in testis) by disrupting transcriptional responses of related genes in the HPG axis. MC-LR causes endocrine disruption with organic and functional damage of the testis.	[60]
MC-LR standard	Male and female Zebrafish	Ultrapathological analysis. Expression in gonad, brain, and liver of growth-related genes in the GH/IGFs axis (*ghrh*, *pacap*, *igf*).	0.3, 3 and 30 µg/L for 90 d	Ultrastructural changes in the liver of male zebrafish were highly consistent with those in females. In gonads of males and females, ultrastructural changes were observed at 3 and 30 µg/L. In both sexes, MC-LR induced a marked concentration-dependent decrease in the expression of brain *ghrh* and *pacap1*. The gene expression in brain *gh* showed a similar downward tendency. In the liver, MC-LR increased *ghrb* expression but decreased the mRNA expression of *igf2a* and *igf2b*. In terms of the transcription of genes in the gonad, there were significant downregulations in the mRNA expression of *ghra*, *igf3*, and *igf2r*. Apparently, alterations in the transcription of related GH/IGFs axis genes were more significant in males than in females.	[59]
MC-LR standard	Male Zebrafish	Pathological analysis and morphometry of testis. Determination of E2 and T testis levels by ELISA. Expression of steroidogenic genes (*CYP*, *StAR*, *17βhsd*) in brain, liver, and testis by RT-PCR. Determination of Erα levels in liver by Western blot.	0.3, 1, 3, 10 and 30 µg/L for 30 d	Dose-dependent testicular damage, characterized by spermatogenesis suppression and the degeneration of interstitial and sustentacular cells. These pathological changes, as symptoms of male reproductive impairment, were consistent with extensive downregulation of brain genes involved in upstream regulation along the HPGL axis. Sex hormone levels and steroidogenesis gene (*CYP*, *StAR*, *17βhsd*) expression in testis exhibited concurrent remarkable increases. The expression levels of testicular genes exhibited stronger positive correlation with E2 and T contents than those in the brain and liver, indicating that MC-LR induced steroidogenesis disruption by primarily affecting the synthesis and autocrine in the testis.	[48]
	H295R cells	Determination of E2 and T testis levels by ELISA. Expression of steroidogenic genes by RT-PCR.	1, 10, 100, 500, 1000, and 5000 µg/L for 48 h	E2 and T contents increased at low concentrations but declined with the increase of MC-LR concentrations. At 1 µg/L MC-LR, the expression of *StAR*, *CYP11A*, *CYP17*, *17βhsd1*, and *17βhsd4* were significantly upregulated. At 10 µg/L, all the genes, except *3βhsd2*, exhibited significant upregulations. At 100 µg/L, *StAR*, *CYP11A*, and *CYP1* remained significantly upregulated. In the range 500-5000 µg MC-LR/L, the expression of all genes gradually declined, with significant down regulations in the expression of *CYP11A*, *CYP19A*, and *17βhsd4*. Increase of E2 content at low doses was positively correlated with extensive upregulation of gene expression along the sex steroidogenesis pathway.	
MC-LR standard	Male zebrafish	Determination of growth and development treatment. Pathological evaluation of testis. Determination of E2 and T serum content. Expression in brain and testis of genes implicated in HPG axis by RT-PCR.	3 and 30 µg/L and NaNO_2_ at 2 and 20 mg/L for 30 d	MC-LR decreased body length. Testis damages increased with increasing MC-LR concentration, and degeneration and lysis of Sertoli cells. Serum E2 levels showed an opposite tendency with a significant concentration-dependent increase in MC-LR exposure groups, with a decrease in nitrite exposure groups. In co-exposure groups, E2 levels presented an increasing trend. Both MC-LR and nitrite significantly decreased T levels; the serum. In the brain: mRNA levels of *gnrh2*, *gnrhr3*, *fshβ*, and *lhβ* decreased in a concentration-dependent manner; *CYP19a1b* gene expression increased. In the testis: a significant downregulation of *ar*, *fshr*, and *lhr*, with a concentration-dependent upregulation in *CYP19a1a*. Significant interactive suppressions between MC-LR and nitrite on the transcriptional levels of testis *ar* and *fshr*.	[61]
*Microcystis aeruginosa*	Female Zebrafish	Histopathological analysis of brain and ovary tissue sections. Determination of E2, T, and VTG plasma levels. Expression in brain, gonads, and liver of *gnrh*, *lh*, and *fsh* genes by RT-PCR.	40 L of *Microcystis aeruginosa* culture for 96 h	Gonadosomatic index decreased. E2 and T plasma levels decreased, while VTG increased. Upregulation of *gnrh*, *fsh*, and *lh* genes.	[62]
MC-LR standard	Female Zebrafish	Histological analysis (liver and ovary). Determination of E2, T, and VTG serum levels. Expression in brain and ovary of genes involved in HPG-axis by RT-PCR. Expression of *17βhsd* and *CYP19a* in ovary by Western blot.	0, 1, 50 µg MC-LR/L for 21 d and depuration for 21 d.	Vacuolation of gonadosomatic tissue. Loss of contact between oocyte and follicular cells. Plasma E2, T, and VTG concentration increased. The expression of mRNA genes involved in the HPG axis (brain and ovary) was altered. Ovarian protein expression (*17βhsd* and *CYP19a*) increased. Recovery of reproduction functions after depuration	[47]
MC-LR standard	Male Zebrafish	Determination of cortisol and glucose serum levels. Expression of different HPI-axis genes in the brain, liver, and kidney by RT-PCR.	1, 5, and 25 µg/L for 30 d	Elevated serum cortisol levels relative to the controls, suggesting that fish suffered continued physiological stress. Conversely, serum glucose levels were significantly decreased. In accordance with increased circulating cortisol levels, the transcription levels of HPI-axis genes were significantly increased.	[63]
MC-LR standard	Female Zebrafish	Observation of death and deformation rates on F1. Histopathological observation. Determination of cAMP and VTG content.	1, 5, and 20 µg/L for 30 d	The F1 zebrafish were prematurely hatched. F1 generation zebrafish showed spinal curvature, pericardial swelling, and reduced pigmentation. Enlargement of the gap between the oocytes and the follicular cell layer in the ovaries after exposure to MC-LR. Decreased VTG and cAMP levels in the 20 µg/L group. MC-LR enhanced the phosphorylation of proteins in the MAPK and MPF pathway.	[64]
*Microcystis aeruginosa*	*Daphnia magna*	Determination of survival, growth, development, and reproduction.	MaE exudates of 2 × 10^4^ cells/mL or 4 × 10^5^ cells/mL densities for 21 d	MaE of high cell density increased body length, eggs production, and the intrinsic growth rate.	[65]
		Determination of VTG, ecdysone, and juvenile hormone levels and 17βhsd activity. Expression in whole body of different genes by RT-PCR.	MaE exudates of 2 × 10^4^ cells/mL or 4 × 10^5^ cells/mL densities for 24, 48, and 96 h	Both treatments increased VTG levels, ecdysone and juvenile hormone, and 17βhsd activity. Increased *vtg1* and *vtg2* genes expression.	
Pure MC-LR and Nodularine-R standard	MELN cell line (transfected from MCF-7)	Determination of estrogenicity and anti-estrogenic activity by MELN assay. Evaluation of cell viability by MTT assay.	2.01 × 10^−9^–6.04 × 10^−8^ M of MC-LR for 24 h and 2.43 × 10^−9^–1.46 × 10^−7^ M of NOD-R	Both toxins with ER-antagonist activity caused significant estrogenic activity. At low concentrations (not cytotoxicity), the induction of the luciferase activity was significantly higher with MC-LR than with NOD-R, indicating that the dose-response effects of MC-LR appear more rapidly than NOD-R. Only MC-LR at high concentrations significantly decreased cell viability.	[42]
MC-LR and -RR, from a purified extract of different cyanobacteria	HaCaT, H4IIE.luc, MVLN, P19/A15, and MDA-kb2 cell lines	Determination of cytotoxicity by neutral red and MTT assay. Evaluation of receptor-mediated effects.	0.006–0.1 g/L for individual MCs and their mixture	Estrogenic potency of some extracts of cyanobacteria was demonstrated, being higher than extracts of single cyanobacterial species. There was no dioxin-like, glucocorticoid, or anti/retinoic activities for any of the extracts studied.	[43]
Extracts and exudates of pure cyanobacterial cultures (*Anabaena, Aphanizomenon*, *Microcystis*)	MVLN transfected with the ER-linked luciferase gene under control of estrogen responsive element	Determination of estrogen receptor-mediated effects.	0.001–0.25 g dw/L for 24 h	Except for *Aphanizomenon flos-aquae*, exudates from all tested pure strains of cyanobacteria were estrogenic. The greatest induction of the luciferase reporter gene was caused by extracts of *Microcystis aeruginosa*. The greatest tested concentrations of exudates from *Aphanizomenon gracile* induced a maximum response of luciferase expression, while the other exudates did not cause a maximum response.	[49]
MC-LR from a purified extract of *Microcystis aeruginosa*	MMV-Luc (estrogen responsive), TARM-Luc (androgen responsive), and TGRM-Luc (glucocorticoid responsive) cell lines	Determination of cytochrome P450-mediated biotransformation of E2 by HLM. Quantification of E2 and biotransformation products by LC-MS/MS.	0.5 and 5.0 µM for 48 h	The major estradiol oxidation products in the HLM assay were 2-hydroxyestradiol and estrone, with only traces of 4-hydroxyestradiol and estriol. The concentrations of 2-hydroxyestradiol following co-incubation of estradiol were significantly lower compared to when estradiol was incubated with HLM alone. In contrast, the concentrations of estrone were relatively higher.	[52]
MC-LR standard	Female SD rats	Determination of MC-LR-protein PP1 in the liver and ovary by Western blot.	200 µg/kg for 6 d	MC-LR could enter ovary.	[66]
	Female BALB/c mice	Determination of GSI. Histological evaluation of follicles. Estrous cycle monitoring. Determination of LH, FSH, E2, and P4 serum levels by ELISA.	5 and 20 µg/kg for 28 d, i.p.	The reduction of GSI indicated a detrimental effect to the female reproductive system. The composition ratio of the number of primordial follicles in the high-dose group decreased. The duration of the proestrus and estrous stage decreased: mice exposed to MC-LR exhibited an abnormal estrous cycle. P4 level decreased without evident changes in FSH, LH, or E2.	
MC-LR from a purified extract from *Microcystis aeruginosa*	Female BALB/c mice	Determination of GSI. Histological evaluation of follicles. Estrous cycle monitoring. Determination of E2 and P4 serum levels by ELISA.	1, 10, and 40 µg/L for 3 and 6 months	The GSI of females significantly decreased. Follicle atresia, and decreased primordial, primary, secondary, and antral follicles. Disorder of estrus cycles (shortened estrus and prolonged distrus). E2 levels decreased while P4 increased (disturb of steroidogenesis).	[67]
	Mouse granulosa cell	Cell viability. Determination of MC-LR by immunofluorescence. LPO and antioxidant enzymes.	1.25, 5, and 20 µM for 48 h	Cell viability decreased. MC-LR was detected. LPO increased after 24-h MC-LR exposure, and CAT and SOD activities decreased.	
MC-LR, -LA, -LY, -LF standards	Female CD-1 mouse	Determination of ovotoxicity in fresh and vitrified follicles. Measurement of E2 levels.	0.1, 1, and 10 µM	MC-LR was the least ovotoxic MC-congener with comparable follicle survival rates at all exposure concentrations, but significantly decreased follicle terminal diameter. MC-LA, LF, and LY decreased follicle survival rates and inhibited follicle growth at 10 μM. MC-LF was found to be the most ovotoxic MC congener and exhibited dose-dependent ovotoxicity. In fresh follicles, follicle development and survival results exhibited comparable ovotoxicities between vitrified and fresh follicles for all MC congeners, suggesting that vitrification provides an efficient and reliable follicle resource for ovotoxicity screening. Follicles treated with MC-LF at 0.1 μM did not affect follicle survival and development. However, the E2 secretion was decreased.	[68]
MC-LR standard	Female SD rats	Histopathology observation of hypothalamus, pituitary, ovary, adrenal, and thyroid gland. Quantification of serum levels of CRH, ACTH, GnRH, T, E2, TRH, FT4, FT3, LH, FSH, and TSH by ELISA. Hypothalamus, pituitary, and ovary mRNA expression of *crh*, *gr*, *gnrh1*, *erα*, *erβ*, *fshr*, *lhr*, *3βhsd*, *17βhsd* by qRT-PCR.	i.p. injections of 36.50, 54.75, or 73.00 μg MC-LR/kg b.w. for 24 h	Occasional shrinkage of neurons and darkened staining of cells in hypothalamus. In the pituitary gland, parenchymal cells lost cytoplasm, with fragmentation and lysis of nuclei. In ovaries, rats exposed to 54.75 or 73 μg MC-LR/kg exhibited hyperaemia, cytoplasmic loss, abnormal nuclear change, and nuclear dissolution. Necrosis of local granular cells at 73 μg MC-LR/kg. Broken nuclei, necrosis of follicular epithelial cells, and reduced intracellular colloid in thyroid gland. Serum concentrations of CRH and ACTH decreased. Less concentrations of GnRH and E2, but greater concentrations of LH, FSH, and T. TRH, fT4, and fT3 decreased, but TSH concentration increased. mRNA for crh and gr were downregulated compared with the control. Expression of gr was downregulated while expression of pomc was up regulated. In the adrenal gland, levels of mRNA for cyp11a1 and 3βhsd were significantly lower. In the hypothalamus, mRNA expressions of gnrh1, erα, and erβ were downregulated. Expression of erα was downregulated while expression of erβ was upregulated. In the ovary, expressions of lhr, erα, erβ, star, cyp11a1, 3βhsd, and 17βhsd were downregulated.	[3]

ACTH: adrenocorticotropic hormone; cAMP: cyclic adenosine monophosphate; CAT: catalase; CRH: corticotropin-releasing hormone; CYP: cytochrome P-450; d: days; E2: 17β-estradiol; 3βhsd: 3β-Hydroxysteroid dehydrogenases; 17βhsd: 17β-Hydroxysteroid dehydrogenases; ER: estrogen receptor; FSH: follicle-stimulating hormone; FT3: free triiodothyronine; FT4: free thyroxine; GnRH: gonadotropin-releasing hormone; GSI: gonadoxomatic index; h: hours; HPG: hypothalamic-pituitary-gonadal; HPGL: hypothalamic-pituitary-gonadal-liver; HPI: hypothalamic-pituitary-interrenal axis; i.p.: intraperitoneal; LC-MS: Liquid Chromatography tandem mass spectrometry; LH: luteinizing hormone; LPO: lipid peroxidation; MAPK: mitogen-activated protein kinases; MaE: *Microcystis aeruginosa* exudates; MTT: 3-(4;5-dimethylthiazol-2-yl)-2;5-diphenyltetrazolium bromide; NOD-R: Nodularine-R; P4: progesterone; RT-PCR: real time polymerase chain reaction; SOD: superoxide dismutase; StAR: steroidogenic acute regulatory protein; T: testosterone; TRH: thyrotropin-releasing hormone; TSH: thyroid-stimulating hormone; VTG: vitellogenin.

### 2.2. Androgenic/Antiandrogenic Effects of MCs and Consequences on Reproductive Toxicity

The androgenic/antiandrogenic effects of MCs and their potential influence on the reproduction of males are shown in Table 2. Most of the studies were carried out in vivo on mammals, while in vitro studies were less frequent, highlighting the research performed with a double strategy, using in vivo and parallel in vitro assays [45,46,71,72,73,74,75,76,77]. Moreover, only a few studies have been performed using fish [78] and, occasionally, the effects of MC-LR have been explored in amphibians [79,80] and crustaceans [81,82]. Almost all of the studies were carried out with the pure standard MC-LR, apart from Chen et al. [78], and, consequently, all of the endocrine disrupting effects observed are due to the toxin and could not be attributed to other active compounds present in purified *Microcystis* extracts.

The first studies demonstrated MC-LR-induced endocrine effects and male reproductive toxicity via both acute and subchronic exposures in mammals [71,83]. MC-LR lowered the T, LH, and FSH serum levels and decreased testis weight and sperm concentration in rats after 28 days of exposure [71], and, similarly, decreased serum T concentrations, impaired sperm quality, and led to testicular injury in mice chronically exposed to low-dose exposures of MC-LR (3–6 months) [83]. Numerous studies have reported a decrease in T levels after exposure to MC-LR in mammals [46,71,72,75,76,77,81,82,83,84]. However, the response in the case of FSH and LH levels or the expression of FSH and LH genes after toxin exposure was more variable: sometimes it decreased [71,72]; at times it increased [46,80,83,84]; and even initial increases were followed by decreases in mice exposed to MC-LR [45,46]. All these changes suggested the potential endocrine toxicity of MC-LR. 

In relation to the distribution of MC-LR in male gonads, the toxin was found in aquatic organisms [85,86] and in fish [87]. In mammals, diverse studies have confirmed the localization of MC-LR in testes [45], and the target cells in testes were explored, such as cultured spermatogonia, Sertoli cells, and Leydig cells (LCs) [45,71,76]. Li et al. [71] investigated the toxicity of MC-LR to cultured LCs, and demonstrated decreased T production and increased reactive oxygen species (ROS) and lipid peroxidation (LPO), showing that oxidative stress could play a role in cell apoptosis of these cells. 

Chen et al. [83] suggested that MCs in mice are transported to the testis via the blood, and that the Leydig cells were the initial target. The LCs have an important role in the synthesis and secretion of androgens, mainly T, which are essential for spermatogenesis and sperm maturation. Moreover, these authors indicated that serum LH and FSH increased in response to MC-LR treatment, especially after 6 months of exposure, and this could be due to the regulation of secretion of T by LH in a negative feedback manner. Consequently, the suppression of T secretion stimulated the release of LH to maintain homeostasis. In addition, the apoptosis of cells, increased in a dose- and time-dependent manner to MC-LR, disrupted the ability of Sertoli cells to secrete inhibin leading to an increased secretion of FSH [83]. 

Chen et al. [84], in rats exposed to i.p. with MC-LR for a long time (50 d), found decreased T levels, while FSH and LH were increased, and morphological alterations of the testes were reported. Thus, the testes index (calculated by the formula: (testes weight/body weight) × 100%) in the group of rats exposed to the highest dose decreased in comparison to the controls, and the space between the seminiferous tubules was increased (light microscope). Ultrastructural observations showed cytoplasmic shrinkage, cell membrane blebbing, swollen mitochondria, and a deformed nucleus. The authors suggested that the cytoskeleton disruption and mitochondria dysfunction induced by MC-LR could interact through ROS formation and resulted in an impairment of the reproductive system. The effects on LCs cells induced by MC-LR were confirmed by Chen et al. [73] after chronic exposure to low concentrations of MC-LR in mice. In this work, the number of LCs in the testes of mice was decreased, while macrophages were significantly increased. By using a co-culture system, they studied the interaction between macrophages and LCs in the presence of MC-LR: stimulation of macrophages to produce TNF-α, and secreted TNF-α induced LCs apoptosis by binding to the tumor necrosis factor receptor 1 (TNFR1) on these cells, and by activating the ROS-p38MAPK signaling pathway. Furthermore, they reported that GAS6 (a protein that binds apoptotic cells and macrophages, creating a bridge) mediated phagocytosis of apoptotic LCs by binding to the Axl receptor on macrophages and phosphatidylserine (PtdSer) on apoptotic LCs. In summary, reduced serum T levels could be associated with decreased LCs due to LCs apoptosis by immune cells after MC-LR exposure in mice. 

In contrast to the suggestion that LCs are a potential cellular target of MC-LR, Wang et al. [88] reported that MC-LR could enter testicular tissues in mammals, and spermatogonia and Sertoli cells are critical target cells of MC-LR, whereas MC-LR was not detected in LCs and had no cytotoxicity on these cells [45,88]. These authors indicated that MC-LR could exert an indirect dysfunction of these cells, a secondary effect due to the damage to the HPG axis caused by the toxin. This hypothesis was supported by the decrease induced by MC-LR in the hypothalamic gonadotropin-releasing hormone (GnRH) expression in a dose- and duration-dependent manner [45].

Similarly, Xiong et al. [72] evaluated the effects of MC-LR on the HPG axis in mice exposed to different concentrations of MC-LR (1–14 d). In this work, the toxin impaired the spermatogenesis of mice, perhaps through the direct or indirect inhibition of GnRH synthesis at the hypothalamic level, which resulted in decreased levels of LH and a suppression of T production in the testes. The in vitro experiments carried out by these authors on LCs confirmed that MC-LR did not affect T synthesis by direct damage of these cells.

As the LCs (the main producers of T) were not injured by MC-LR in some experiments, Wang et al. [46] demonstrated that the GnRH neurons were the target of MC-LR in rats. In this work, serum levels of GnRH, LH, FSH, and T showed a similar pattern of early-stage increases and late-stage declines. The authors indicated that there might be two mechanisms by which MC-LR affected the hypothalamic-pituitary axis: (1) MC-LR could attack multiple targets along the axis, and, consequently, some of the targeting led to decreased transcription of GnRH while others led to disrupted pituitary activity; (2) MC-LR only affected a single target, such as the GnRH neurons, affecting the expression and secretion of GnRH. Later, the same group investigated the toxic effects of MC-LR on GnRH neurons in the hypothalamus [75] and demonstrated that the toxin could enter GnRH neurons and inhibit GnRH synthesis, resulting in the decrease of serum GnRH and T levels in mice. In vitro, in GT1-7 cells, they also associated this inhibitory effect on GnRH synthesis with the activation of the cyclic adenosine monophosphate (cAMP/protein kinase A (PKA)/cAMP response element-binding protein (CREB)/c-Fos. Furthermore, they found that miR-329-3p was significantly reduced in GT1-7 cells after MC-LR exposure, and the toxin induced PKA activation by regulating the expression of PRKAR1A and PRKACB at the post-transcriptional level. These data contribute to understanding, at the molecular level, male infertility induced by MC-LR because of dysfunction of the HPG axis. 

With the aim of studying the specific mechanisms of the uptake of MC-LR by GnRH-secreting neurons, Ding et al. [89] demonstrated in vitro, in GT1-7 cells, that at least four organic anion transporting polypeptides (*Oatp1a4, Oatp1a5*, *Oatp5a1*, *Oatp2b1*) were expressed in GnRH neurons at the mRNA level, but only *Oatp1a5* was expressed at the protein level. They also reported that MC-LR could not be transported into Oatp1a5-deficient GT1-t7 cells, which were protected against the effects of the toxin. Later, the same authors confirmed in vivo that, after MC-LR exposure, mice exhibited decreased GnRH levels [74]. Furthermore, in GT1-7 cells, the toxin stimulated intracellular Ca^2+^ and cAMP to activate the protein kinase c (PKC), PKA, and MAPK signaling pathways in GnRH neurons. Then, the toxin altered some protein levels and changed the activity of GnRH transcription factors, such as Pbx1a, Oct-1, Dlx-2, Otx-2, c-Jun, and c-Fos. All these data support the conclusion that MC-LR can decline the synthesis of GnRH in GnRH neurons via the Ca^2+^-PKC-NF-kB-cAMP-PCA-Creb and Erk/P38-MAPK signaling pathways. Recently, Jin et al. [77], using a combination of in vitro (the same GT1-7 cells) and in vivo experiments (male mice), demonstrated that MC-LR could activate the endoplasmic reticulum stress (ERs), leading to apoptosis of GnRH neurons and decrease GnRH synthesis, and reduce the secretion of T. Moreover, pre-treatment with a ERs inhibitor reduced the apoptotic rate on cells, whereas apoptotic cell death was increased by pre-treatment of GT1-7 cells with an autophagy inhibitor. Autophagy is a protective cellular process, activated by ERs stress and could potentially protect cells from apoptosis induced by MC-LR. 

In contrast to previous studies, which suggested that LCs are an indirect target of MC-LR [45,74], a recent study carried out in vivo in mouse testes and in vitro in TM3 cells [76] indicated that MC-LR can enter and accumulate into mouse testes and in TM3 cells, and provided results indicative that LCs are a direct target of MC-LR. The main findings of this work were: (1) MC-LR reduced serum and testicular T levels; (2) MC-LR downregulated steroidogenic proteins and synthesis in both models; (3) MC-LR evoked GCN2/elF2α signaling in TM3 cells; (4) GCN2iB, a specific inhibitor of GCN2 signaling, attenuated MC-LR-induced eIF2α phosphorylation and subsequent downregulation of steroidogenic proteins; (5) pre-treatment with N-Tert-Butyl-α-Phenylnitrone (PBN), a free radical scavenger, reduced the activation of GCN2/elF2α and the downregulation of steroidogenic proteins in TM3. All these results indicated that MC-LR at least partially inhibited T synthesis via the ROS-mediated GCN2/elF2α pathway.

In the case of fish, only one study performed on tilapias (*Nile tilapia*) demonstrated the effects of MCs extracted from lyophilized cells of *Microcystis aeruginosa* and purified MC-LR on endocrine endpoints and reproduction. After 28 d of exposure, T levels increased in *Microcystis* and MC-LR-exposed fish, and interferences with the expression of genes involved in the HPLG axis and balance of sex hormones were also induced [78]. 

Endocrine-disrupting effects induced by MC-LR have also been investigated in male frogs (*R. nigromaculata*) [79,80]. MC-LR induced toxic effects on the reproductive system of frogs, decreased T levels, and increased E2 contents. After prolonged exposure, the relative expression levels of P450 aromatase (*CYP19A1*) and steroidogenic factor 1 (*SF-1*), important factors in reproductive toxicity in males by mediating molecular regulatory mechanisms, were enhanced [79]. Later, these authors [80] confirmed decreased T and increased E2 concentrations after exposure to 1 and 10 µg/L MC-LR for 14 d. The toxicity mechanisms proposed in amphibians were: (1) the downregulation of *HSD17B3* gene expression caused the disruption of T synthesis; (2) the upregulation of *CYP19A1* gene expression directly stimulated conversion of T to E2; (3) this upregulation of *CYP19A1* stimulated conversion of androstenedione to estrone (E1) and the upregulation of *HSD17B1* gene expression stimulated conversion of E1 to E2. Moreover, the decrease of T and abnormal gene expression of the AR and oestrogen receptors (ESR1) caused interferences in spermatogenesis in frogs [80]. In the case of the giant freshwater prawn, *M. rosenbergii* [81], results showed that MC-LR could disrupt the testicular development of *M. rosenbergii*, perhaps by affecting T levels (decreased) and the expression of gonadal developmental-related genes in the testes and eyestalk. Moreover, testicular germ cells, mitochondria, and cell junctions were also damaged. In aquatic crustaceans (*M. nipponense*), the parental transference of MC-LR induced testicular dysfunction and reproductive and offspring immune changes in exposed prawns [82]. MC-LR-damaged testicular germ cells, reduced T levels in the hemolymph, and inhibited the development of the testes. The F1 offspring showed a downregulation of immunity molecules and antioxidant enzymes, and higher expression levels of apoptotic-related genes. The mechanisms involved could be the induction of mitochondrial apoptosis of the F1 offspring after continuous exposure to MC-LR.

Moreover, changes in androgenic biomarkers have also been observed in females. Thus, some studies, as included in Table 1, demonstrated reproductive effects in female zebrafish after exposure to MC-LR, with changes in several hormones (e.g., T levels) which modulate reproductive toxicity, as well as changes in the transcription of HPG-axis genes [41,44,47,58]. In female rats, acute exposure to MC-LR affected histopathology and induced changes in several hormones in the HPG axis (GnRH, LH, FSH, T, and E2), and in the transcription of genes for hormone synthesis (as well as in the case of the HPA and HPT axes) suggesting endocrine-disrupting effects [3].

In summary, reports have shown that MC-LR can show androgenic effects mediated by different mechanisms, although cellular targets need to be still defined (LCs, GnRH neurons, etc.). Moreover, in this case, differential responses between pure MC-LR, and other congeners have not been explored. Furthermore, further cyanobacterial blooms/extracts investigations would be of interest, as they represent a more real exposure scenario. 

**Table 2:** Androgenic/antiandrogenic effects of MCs and consequences on male reproductive toxicity.

Toxin	Experimental Model	Assays Performed	Concentration Ranges and Exposure Conditions	Main Results	References
Pure MC-LR standard	Male SD rats	Sperm analysis. Determination of T, LH, and FSH serum levels.	5, 10, and 15 µg/kg b.w. for 28 d	Decrease of epididymal sperm concentration and motility. Slight testicular atrophy, slight deformation of androgonial and spermatogenic cells, and reduced numbers of Sertoli cells and mature sperm. Decreased T, LH, and FSH serum levels.	[71]
	Leydig cells	Cell viability by MTT. Measurement of apoptosis. Determination of T concentration and ROS production. Detection of LPO and SOD activity.	0.5, 5, 50, and 500 nM for 12, 24, and 48 h	Reduced cell viability. Decrease of T content. Increase of ROS and LPO, reduced cytosolic SOD activity.	
Pure MC-LR standard	Male SPF mice	Sperm morphology. Determination of T, LH, and FSH serum levels. Histopathological evaluation of testis. TUNEL assay for detection of apoptotic cells in testis.	1, 3.2, and 10 µg/L for 3 and 6 months	Sperm motility decreased with 3.2 and 10 µg/L. Serum levels of T decreased, whereas FSH and LH levels increased. At the highest MC-LR level: testicular atrophy and derangement of spermatogenic cells. Six-month results showed a significant increase of apoptotic cells.	[83]
Pure MC-LR standard	Male BALB/c mice	Expression in hypothalamus, pituitary, and testis of *gnrh*, *lh* and *fsh* genes by RT-PCR. Determination of T, LH, and FSH serum levels.	3.75, 7.5, 15, and 30 µg/kg b.w. for 1, 4, or 7 d	Expression of *gnrh* gene was downregulated. *fshβ* and *lhβ* genes were upregulated at 1 and 4 days; however, its expression was downregulated at 14 days. Serum levels of T, LH, and FSH increased at 1 and 4 days, and decreased at 14 days.	[45]
	Leydig cells	Determination of MC-LR uptake. Cell viability by CCK-8 test. T concentrations.	1, 10, 100, 250, 500, 750, and 1000 nM for 2 h	MC-LR was not able to enter Leydig cells. There were no statistically significant differences in cell viability and T concentration.	
Pure MC-LR standard	Male Wistar rats	Histology of testis. Mitochondrial swelling and DNA damage. Determination of T, LH, and FSH serum levels. Determination of ROS production. Expression in testis of different cytoskeletal and mitochondrial genes by RT-PCR.	1 and 10 µg/kg for 50 d	Testis index decreased (10 µg/kg group). Blockage in seminiferous tubules was observed and spermatogonia showed apoptotic characters and cytoplasmic shrinkage. DNA fragmentation was observed. Serum levels of LH and FSH increased, while T levels decreased. ROS production was increased. The expression of all mitochondrial genes was increased, whereas some cytoskeletal genes decreased (β-actin, β-tubulin, Radixin) and some increased (vimentin, ezrin, moesin).	[84]
Pure MC-LR standard	Male C57BL/6 Mice	Determination of T, FSH, and LH serum levels. Evaluation of epididymal sperm. Expression of *gnrh*, *lh* and *fsh* genes in hypothalamus, pituitary, and testis by RT-PCR.	3.75, 7.5, 15, 30 µg/kg b.w. for 1, 4, 7, and 14 d	Significant reduction in epididymal sperm production. T and LH production was inhibited. Reduction of *lh* and *gnrh* genes expression.	[72]
	Leydig cells	Cell viability and detection of T levels.	1, 10, 100, 250, 500, 750, and 1000 nmol/L for 48 h	No significant differences on cell viability and T production	
Pure MC-LR standard	Male SD rats	Detection of GnRH, FH, LSH, and T serum levels. TUNEL assay for detection of apoptotic hypothalamic cells. Hypothalamus *gnrh1*, *lhβ*, and *fshβ* detection by RT-PCR. Western blot and immunofluorescence for MC-LR detection in GnRH neurons.	30 µg/Kg body weight for 1, 3, 5, 7, and 14 d	Serum levels of GnRH, LH, FSH, and T showed a similar pattern of early-stage increase and late-stage decline. Remarkable apoptotic hypothalamic cells after 7 and 14 days of exposure. MC-LR could be observed in GnRH neurons.	[46]
	GT1-7 cells	Immunocytochemistry for MC-LR detection. Cell viability, LDH rate, and GnRH concentration. Measurement of the concentrations of cAMP, Ca^2+^, and AC activity by ELISA.	1, 10, 100, 1000, and 10,000 nM for 48 h	MC-LR could be taken up by GT1-7 cells. Cell viability decreased and LDH release increased. A concentration-dependent increase of cAMP, Ca^2+^, and AC activity.	
Pure MC-LR standard	Male BALB/c mice	Determination of macrophages enrichment in testes by flow cytometry. Expression in testis of *Hsd3β*, *Hsd17β*, *StAR*, *TNF-α*, *IL-6* and *MCP-1* genes by qRT-PCR. Western blot, coimmunoprecipitation, and ELISA immunohistochemical analysis. Analysis of testicular tissues by immunofluorescence.	1, 10, 20, and 30 µg/L for 180 d	At 20 and 30 µg/L, the number of Leydig cells, mRNA levels of *StAR*, *Hsd3β*, and *Hsd17β* decreased. Protein levels of *Hsd3β* decreased at 10 µg/L. Immunohistochemical staining assay demonstrated an increase of macrophages in testes and flow cytometry identified that MC-LR could modulate the number of testicular macrophages. Levels of *TNF-α*, *IL-6*, and *MCP-1* increased. Leydig cells apoptosis was drastically increased when co-cultured with macrophages in the presence of MC-LR. mRNA levels of *TNF-α* increased in a time-dependent manner in macrophages, peaking at 3 h post exposure. *GAS6* mRNA levels increased at 24 h, and *GAS6* cytokine levels declined in medium at 6 h. After addition of MC-LR into the co-culture system of macrophages and LCs, no marked increase of *GAS6* in the cytoplasm of LCs.	[73]
	RAW264.7 cells	Determination of macrophage polarization by flow cytometry.	500 nM for 24 h	Apoptosis in LCs increased in a time-dependent manner when LCs were cultured with MC-LR. Moreover, TNF-α levels in macrophages were significantly elevated at mRNA and protein levels. Phosphorylation levels of NF-κB were upregulated in macrophages.	
Pure MC-LR standard	Male BALB/c mice	MC-LR uptake in GnRH neurons by immunofluorescence. GnRH and T levels in serum by Western blot.	20 µg/Kg body weight for 7 d	GnRH mRNA in hypothalamus and the content in serum decreased. Immunofluorescence staining in hypothalamus tissue sections showed the presence of MC-LR in the cytoplasm of GnRH neurons.	[74]
	GT1-7 cells	GnRH synthesis and secretion by RT-PCR. Intracellular levels of cAMP and Ca^2+^ by ELISA. Determination of Ca^2+^ and cAMP by ELISA. Measurement of soluble GnRH by HPLC-MS/MS.	10, 50, 100, 500, and 1000 nM for 1, 3, 6, 12, 24, and 48 h	MC-LR can be transported into GT1-7 cells. Declination of *gnrh* mRNA levels, and increase followed by a decrease of GnRH releasing. Intracellular levels of cAMP and Ca^2+^ increased. PKC kinase activity enhanced with the time of MC-LR exposure, meanwhile, a time- and concentration-dependent increasing trend of phosphorylation levels of PKC and NF-κB was observed. PKA kinase activity enhanced with the time of MC-LR-exposure, and Prkacb (PKA catalytic subunits) and the phosphorylation of Creb increased with the time and concentration of MC-LR. The toxin enhanced the phosphorylation of downstream proteins in the MAPK pathway, such as the phosphorylation of *Erk* and *p38*. mRNA levels of *Oct-1, Dlx-2, Otx-2* decreased, and *c-Fos* and *c-Jun* increased gradually. Their protein expressions showed similar trends.	
Pure MC-LR standard	Male ICR mice	Determination of GnRH concentration in the brain by immunofluorescence. Determination of GnRH serum levels by HPLC-MS. Determination of T serum levels by ELISA.	1, 7.5, 15, and 30 µg/L for 180 d	GnRH and T serum levels decreased.	[75]
	GT1-7 cells	Cell viability. Determination of GnRH levels. Determination of cAMP levels by ELISA. Determination of PKA kinase activity.	1 mg/mL for 0.25, 0.5, 1, 3, and 6 h	Cell viability and GnRH levels decreased. cAMP levels increased. Upregulation of PKA activity.	
Pure MC-LR standard	Male ICR mice	Sperm count. T measurement by radioimmunoassay. Immunohistochemistry of testis. Immunoblotting of testis. Detection of MC-LR.	Daily i.p. injection of 20 µg/kg for 35 d	Serum and testicular T levels decreased. MC-LR located in both Leydig cells and seminiferous tubules. Testicular steroidogenic proteins (*StAR*, *CYP11A1*, and *CYP17A1*) downregulated.	[76]
	TM3 cell line	Cell viability. Detection of MC-LR. ROS detection.	0.05–20 µM MC-LR for 6–24 h	MC-LR was increased in a concentration-dependent manner in MC-LR-treated TM3 cells. Concentrations of MC-LR used did not affect viability. p-GCN2 and p-eIF2α increased at 24 h. Intracellular ROS began to increase at 6 h and continued to rise at 12 h and 24 h. Pre-treatment with PBN alleviated MC-LR-induced activation of GCN2/eIF2α signaling in the cells.	
Pure MC-LR standard	Male BALB/C mice	Immunohistochemistry of the brain. Apoptosis detection by TUNEL. Serum levels of T and GnRH by ELISA. Electron microscopy of the brain.	1, 7.5, 15, and 30 μg/L 180 d	Serum T and GnRH declined. GnRH levels were downregulated in the brain. Remarkable apoptotic cells in hypothalamus. Hypothalamus tissues displayed increased expression of active-caspase3. Hypothalamus neuron cells showed distinct ultrastructure changes.	[77]
	GT1-7 cell line	Cell viability. Analysis of cell cycle. Cell apoptosis by flow cytometry. ROS content detection. Measurement of intracellular Ca^2+^ mRNA expression of *gnrh* by qRT-PCR.	50, 500, and 1000 nM	GnRH protein released into the supernatant decreased. mRNA levels of *gnrh* were remarkably reduced. MC-LR induced cell cycle dysregulation, apoptosis, and inhibited cell growth. Generation of ROS increased. MC-LR was able to cause the release of intracellular Ca^2+^. Pre-treatment with an endoplasmic reticulum stress inhibitor, such as 4-phynyl butyric acid, reduced apoptosis rate in the cells. The pre-treatment with the autophagy inhibitor 3-MA could increase the apoptosis caused by the toxin.	
MC-LR from a purified extract from *Microcystis aeruginosa* and MC-LR standard	Male *Nile tilapia*	Determination of E2 and T serum levels. Expression in brain, gonads and liver of different genes in HPGL axis by RT-PCR.	9.6 µg/L for 28 d	Serum E2 and T levels increased. Transcripts of 13 genes (*GHRH*, *PACAP*, etc.) were altered.	[78]
Pure MC-LR standard	Male *Rana nigromaculata*	Determination of sperm motility and deformity. Testes histopathology by electron microscopy. Quantification of E2 and T serum levels. Analysis of SF-1 and P450 aromatase protein expression by commercial kit.	0.1, 1, and 10 µg/L for 7 and 14 d	The number of sperm cells and sperm motility decreased in all groups. The sperm abnormality rate was significantly increased. Frogs manifested ultrastructural changes in a concentration-dependent manner. Testosterone content was significantly decreased in all groups, while estradiol content was significantly increased. The protein content of p450 aromatase and SF-1 significantly increased.	[79]
Pure MC-LR standard	Male frogs (*Rana nigromaculata*)	MC-LR concentration in testes by HPLC-MS. Determination of E2, T, LH, and FSH serum levels. Evaluation of cAMP content in testes by ELISA. Expression in brain and testis of genes implicated on the HPG axis by qRT-PCR.	0.1, 1, and 10 µg/L for 7 and 14 d	MC-LR in the testes increased in a concentration- and time-dependent manner. Serum levels of T decreased, while E2 and FSH increased. cAMP content decreased at 7 d but significantly increased at 14 d. mRNA levels of *StAR*, *CYP11A1*, *CYP17A1*, and *CYP19A1* were upregulated, whereas *HSD3B2* and *HSD17B3* levels were downregulated. mRNA levels of AR and ESR1 were upregulated.	[80]
Pure MC-LR standard	*Macrobrachium rosenbergii*	Immunolocalization of MC-LR in testis. Histopathological examinations. Determination of T serum levels. TUNEL assay for determination of testicular germ cells apoptosis. Expression in testis and eyestalk of gonadal-development-related genes (*hsp70*, *sox9*) by RT-PCR.	0.5 and 5 µg/L for 1, 2 or 3 weeks	MC-LR was found in testis, spermatocytes, and Leydig cells. T serum levels decreased. Inhibitory effect on testis development, and testicular germ cells apoptosis. *Hsp70* was upregulated.	[81]
Pure MC-LR standard	Male parental and F1 embryo *Macrobrachium nipponense*	Serum concentrations of T and E2. Transmission electron microscopy of testis. Determination of sperm quality. Measurement of testicular antioxidant ability. Detection of apoptotic cells by TUNEL assay. F1 mRNA expression of different genes associated with apoptosis, immune function, and antioxidant capacity by qRT-PCR and protein expression by Western blot.	0.5 and 5 µg/L for 28 d	T serum levels decreased, but elevated serum E2 levels were found. After 4 weeks, the testes of male prawns showed disturbed development. Increased percentages of spermatogonia (SPG) and spermatocytes (SPC). ROS levels of testis in F0 generation prawns increased, with increased testicular SOD and CAT activities. In contrast, activities of GPx1 and GST decreased. MDO contents increased but GSH content decreased. Treatment with increasing MC-LR concentrations resulted in increased levels of the toxin in the testis of F0 and F1 embryos. Sperm DNA damage and apoptotic cells. Malformation rates in F1 larvae from MC-LR-treated F0 males significantly increased. Lower mRNA levels of *Mn-SOD*, *GPx1*, and *CAT*. Transcript abundances of apoptosis-related genes (Caspase-3 (*Casp3*), caspase-9 (*Casp9*)) were higher. Protein levels of Casp3 and p53 increased in F1.	[82]
Pure MC-LR standard	GT1-7 cells	Cell viability by CCK-8 test. MC-LR uptake by Western blot. Expression of *gnrh* and *OATPs* genes by RT-PCR. Determination of OATPs proteins expression by Western blot. Measurement of soluble GnRH by HPLC-MS/MS.	10, 20, 50, 100, 200, 300, 400, 500, 750, 1000, and 50,000 nM for 48 h	Cell viability gradually decreased. MC-LR can be transported into GT1-7 cells. *Gnrh* transcription levels decreased with increasing MC-LR concentrations. *Oatp1a4*, *Oatp1a5*, *Oatp5a1*, and *Oatp2b1* genes were expressed in GT1-7 cells. Only *Oatp1a5* protein was expressed.	[89]
	OATP1a5-KO GT1-7 cells	Expression of OATPs genes by RT-PCR. Determination of OATPs proteins expression by Western blot. Cell viability by CCK-8 test.	500 nM for 48 h	Pattern of *Oatp1a4*, *Oatp5a1*, and *Oatp2b1* mRNA expression in the Oatp1a5-KO GT1-7 cells was the same as in the intact cells. Like the intact cells, Oatp1a5-KO cells did not express *Oatp1a4, Oatp5a1*, or *Oatp2b1* at the protein level, evidenced by Western blot assay. Cell viability still unchanged.	

AC: adenylate cyclase; cAMP: cyclic adenosine monophosphate; CAT: catalase; c-KIT: stem cell factor receptor; cDNA: deoxyribonucleic acid; E2: 17β-estradiol; eIF2α: eukaryotic translation initiation factor 2α; FSH: follicle-stimulating hormone; GAS6: growth arrest-specific 6; GCN2: general control nonderepressible 2; GPx1: glutathione peroxidase 1; GnRH: gonadotropin-releasing hormone; GSH: glutathione; GSI: gonadosomatic index; hpf: hours post-fertilization; HPG: hypothalamic-pituitary-gonadal; HPGL: hypothalamic-pituitary-gonadal-liver; HPI: hypothalamic-pituitary-interrenal axis; HPLC-MS/MS: High-performance liquid chromatography–tandem mass spectrometry; *hsp*: heat shock proteins; IL-6: interleukine 6; i.p.: intraperitoneal injection; Kg b.w.: Kg per body weight; LC-MS: Liquid Chromatography- mass spectrometry; LDH: lactate dehydrogenase; LH: luteinizing hormone; LPO: lipid peroxidation; 3-MA: 3-methyladenine; MAPK: mitogen-activated protein kinases; MCP-1: monocyte Chemoattractant Protein-1; MDO: malondialdehyde; Mn-SOD: manganese suporoxide dismutase; MTT: 3-(4;5-dimethylthiazol-2-yl)-2;5-diphenyltetrazolium bromide; OATP: organic anion transporting polypeptide; Oct-1: organic cation transporter 1; PBN: N-Tert-Butyl-α-Phenylnitrone; pepck: phosphoenolpyruvate carboxykinase; PI: propidium iodide; PKA/C: protein kinase A/C; pomc: pro-opiomelanocortin; PP1/2A: protein phosphatases 1 and 2A; ROS: reactive oxygen species; RT-PCR: real time polymerase chain reaction; SF-1: steroidogenic factor 1; SOD: superoxide dismutase; SOX9: SRY-Box Transcription Factor 9; SD: Sprague-Dawley; SPF: specific pathogen free; StAR: steroidogenic acute regulatory protein; T: testosterone; TNF-α: tumor necrosis factor-α.

### 2.3. Effects of MCs on Steroidogenesis

Steroidogenesis is the process by which steroid hormones are synthesized from cholesterol. There are two principal pathways of synthesis, the adrenal and the gonadal pathways, and, in both cases, numerous enzymes are involved [2,80]. Thus, the steroidogenic acute regulatory protein (StAR), cytochrome P450 cholesterol side chain cleavage enzyme (*CYP11A1*), cytochrome p450 17 α-hydroxylase/17,20hydroxylase (*CYP17A1*), 3ß-hydroxysteroid dehydrogenase type 2 (*HSD3B2*), *HSD17B3*, and cytochrome P450 aromatase (*CYP19A1*) are important steroidogenic enzyme profiles along the HPG axes in testes [2,80]. Pavagadhi et al. [90] found, in zebrafish exposed to MCs for 30 d, that cholesterol synthesis was significantly affected, suggesting that MCs can cause endocrine disruption even under sublethal conditions; however, separately from cholesterol synthesis, various metabolic pathways, such as essential fatty acids and lipid oxidation, were also perturbed. Several studies included in Table 1 [48,58] and Table 2 [63,76] show the capacity of MC-LR to interfere on the expression of genes involved in steroidogenesis. 

As mentioned above (Section 2.2.), a combined in vitro an in vivo approach is a useful way by which to understand the activities of endocrine disruptors, and both methods were employed by Hou et al. [48] to understand the estrogenic effects of MC-LR via stimulating the steroidogenesis. On the one hand, the effects of MC-LR (1–5000 µg/L) on steroidogenesis were assessed in vitro, in the H295R cells after 48 h of exposure, and the levels of E2 and T increased in a non-dose dependent manner, which showed positive correlations with the expression of steroidogenic genes. Induction of E2 via interference with steroidogenic enzymes, such as an aromatase, the rate-limiting enzyme converting T to E2, is the mechanism suggested for estrogenicity of MC-LR, separately from binding ER [91]. On the other hand, the authors investigated the in vivo effects of MC-LR on male zebrafish exposed to the toxin (0.3–30 µg/L MC-LR) for 30 d. Similar to the in vitro results, sex hormone levels and steroidogenesis gene expression in zebrafish increased, and the critical estrogen threshold was established between 1 and 30 µg/L MC-LR, which is considered environmentally relevant. In this study, E2 and T contents in the testis increased, and upregulation of some steroidogenic genes, especially *CYP19a*, was reported. In the liver, the *vtg1* gene was upregulated, while the transcriptional and protein levels of the ER were decreased. All these results indicated that the estrogenic effects of MC-LR were non-dose dependent, which may result from steroidogenesis stimulation via a non-ER-mediated pathway [48]. These authors indicated that the cyclic chemical structure of MCs is not like other EDCs (nonyl phenol, bisphenol A), and its hydrophilic feature reduces the possibility of it functioning as an ER ligand. Thus, MC-LR is unlikely to cause estrogenic responses via binding ER, and they suggested that MC-LR induces E2 increases via stimulating steroidogenesis rather via the ER-mediated pathway.

Moreover, in vitro MC-LR disturbed steroidogenesis of primary cultured ovarian granulosa cells, responsible for the production of E2 and P4. These cells could uptake MC-LR and should be the target of the toxin, which induces oxidative stress [92]. The ovarian granulosa cells (GCs), essential for the growth and development of follicles, were also exposed to MC-LR for 48 h, and microRNAs (miRNAs) and mRNAs microarray technologies were applied. Numerous miRNAs and mRNAs significantly changed in the treated MC-LR group, and functional analysis indicated that they were involved in proliferation, apoptosis, immunity, metabolism, etc., and, consequently, interferences with the normal function of the cells were reported. Disorders of estrogen and progesterone, and premature ovarian failure (POF) are important factors of infertility, while changes in GCs function are crucial for the induction of infertility [92].

The effects of MC-LR on steroidogenic proteins and GCn2/eIF2α signaling in Leydig cells [76] have been previously commented on (Section 2.2.). In this study, ROS-mediated GCn2/eIF2α activation could explain, in part, the downregulation caused by MC-LR on steroidogenic proteins and synthases. 

An optimized vitrification protocol method to cryopreserve murine immature follicles has been developed and it has been used as a screening method by which to investigate the ovotoxic response to different MCs congeners [68]. The dose-response ovotoxicity screening results revealed that MC-LF was the most ovotoxic MC variant. Moreover, MC-LR, compared to MC-LA, MC-LF, or -LT, showed the least adverse impacts on follicle survival and development, indicative that these MCs variants could cause follicle damage through other molecular mechanisms, different to the inhibition of PP2A. For example, the expression profile and function of OATPs in ovaries could be involved. This screening also demonstrated that MC-LF at 0.1 µM significantly inhibited E2 secretion, indicating that the human-relevant exposure of MC-LF may interfere with ovarian steroidogenesis and exert endocrine disruptive effects [68].

In fish, the endocrine system consists of three main pathways, including the HPG, HPT axis, and HPI axis. Several studies have indicated that the effects of EDs on an endocrine axis pathway could indirectly influence the other axes in fish. Thus, it has been demonstrated that MC-LR induced endocrine disruption, through endocrine axes, perhaps due to interference with steroidogenesis, including cholesterol, cortisol, and estrogens, and, subsequently, resulted in a disruption of glucose homeostasis [63]. Furthermore, zebrafish have also been chosen as a good experimental model in which to investigate the effects of pure MC-LR standard on the HPI axis during early embryonic development (embryos/larvae), 4–168 hpf [93]. At the highest concentration assayed, MC-LR induced higher cortisol concentrations, and the expression of genes along the HPI axis and mineralocorticoid receptor (MR-) and glucocorticoid receptor (GR-) were also changed. This was the first study at the molecular level focused on the endocrine-disrupting effects of MC-LR, including the neurocrine, steroidogenic, and receptor signaling pathways.

Further research is required to confirm the mechanisms involved in the effects of MC-LR on the production of E2, T, and all the key enzymes for steroidogenesis, by using the double strategy of in vitro and in vivo assays, following international guidelines. In the case of steroidogenesis, the use of the in vitro assay OECD TG 456, not applied so far for cyanotoxins, is encouraged. The same would apply for other minority congeners (as variant-specific effects have been reported) and cyanobacterial blooms.

### 2.4. Thyroid Endocrine Disruption of MCs

The different studies focused on the induction of thyroid endocrine disruption of MCs are shown in Table 3. All of them have been performed using the pure standard MC-LR congener, except for one study, that investigated the effects of MC-RR on zebrafish (0.3–3.0 mg/L for 96 hpf) [94]. 

Several studies have reported that subchronic exposure to high MC-LR concentrations can cause thyroid dysfunction in fish [95,96] and in mice [97,98]. Previous studies demonstrated that MCs induced changes in thyroid hormones (THs) production in fish [71], and, since this work, most experiments have been performed using zebrafish embryos or juvenile zebrafish as a model to study the thyroid-disrupting toxicity of MCs [94,96,99,100,101]; although, in other studies, adult zebrafish have been employed [95,102,103]. In mammals, studies are very scarce [3,97,98]. Moreover, in *Xenopus laevis* tadpoles exposed to an ecyanobacterial biomass containing MC-LR, no changes in thyroid-stimulating hormone (TSH) were detected after 21 days of exposure [104].

The thyroid hormone receptor-α is an inducible ligand-activated transcription factor and thyroid hormones exert their physiological effects by binding to them on the HPT axis [105]. In zebrafish larvae exposed to 100–500 µg MC-LR/L, body growth retardation associated with significant decreases in mRNA expression of iodothyronine deiodinase 2 (Dio2), as well as decreased T4 and T3 levels, were reported [99]. These findings indicated that MC-LR could alter gene expression in the HPT axis, which might contribute to MC-LR-induced thyroid disruption [99]. 

Similarly, Liu et al. [95] indicated that MC-LR, at relevant environmental concentrations, resulted in the disturbance of TH homeostasis by disrupting the synthesis and conversion of THs: altered Dio activities and decreased levels of T3. Iodothyronine deiodinases play crucial roles in the mechanisms of thyroid hormone biotransformation and metabolism in peripheral tissues, and are important regulators of circulating and intracellular THs levels in fish [106]. Moreover, hyper-stimulated THs synthesis and secretion, elevated T4 levels, as well as an upregulation of genes involved in THs synthesis could be considered as negative feedback from the hypothalamus and pituitary due to the decreased levels of T3. All these lead to a hypothyroidism state after exposure to this toxin. 

Similar effects were also reported in vivo in juvenile Chinese rare minnows exposed to MC-LR for 7 d [107], with alterations in Dio activities that resulted in decreased T3 production. The authors indicated that this decrease could be due to a reduction in the rate of conversion of T4 to T3 because of the reduced activity of Dio2. Moreover, the reduction of Dio3 activity associated with the downregulation of mRNA expression in Dio3 could reflect a negative feedback compensatory mechanism against decreases in T3 concentrations in the exposed fish. The sodium/iodide symporter (NIS) gene is known to be involved in thyroid hormone synthesis, and changes in its expression may alter thyroid hormone production. The down-regulation of NIS expression indicates that MC-LR could induce thyroid disruption. Moreover, the transport protein for thyroid hormones transthyretin (TTR), mainly secreted by the liver, regulates the supply of the various thyroid hormones in target tissues. The authors concluded that the changes in transcription of the NIS, TTR, thyroid hormone receptor-α, and iodothyronine deiodinase genes indicated disturbances in thyroid hormone synthesis, transport, and metabolism of fish, leading to a decline in either T4 or T3 production. In agreement with previous work [95], the hypertrophy of thyroid follicle epithelial cells could be secondary to the negative feedback regulation because of the lowered thyroid hormones, also resulting in hypothyroidism in this juvenile fish species.

In adult zebrafish, the effects of MC-LR on the THs homeostasis were also investigated after longer exposure (1–28 d) to environmental concentrations (1–25 µg MC-LR/L) [102]. In contrast to previous experiments in juvenile zebrafish [95], in adult zebrafish, no differences were found in the histopathology of thyroid follicles and T4 levels were unchanged, confirming that exposure to MC-LR did not inhibit the production of THs in adults. However, in agreement with juvenile zebrafish exposed to the same MC-LR concentrations, a significant decrease in T3 levels associated with decreased Dio2 activity in male zebrafish was observed, suggesting the important role of Dio2 in the disturbances of thyroid hormones. Moreover, the mRNA expression of TSH, TTR, and TRS appeared to be a dynamic process, as expression first decreased and then increased with continued exposure. The authors suggested that although MC-LR exposure can alter the metabolism of THs, fish can trigger compensatory mechanisms to maintain THs homeostasis [103]. 

This fact has also been confirmed in adult zebrafish exposed to acutely higher concentrations of MC-LR (50–400 µg MC-LR/L) during 24, 48, 72, or 96 h, which disrupted the thyroid hormone metabolism by altering Dio activity and gene expression of the HPT axis [103]. These authors confirmed that MC-LR induced a negative feedback regulation of the HPT axis in adult zebrafish, as the females were more sensitive than the males. Notably, it was reported that these changes may affect the complement system through the regulation of c9 mRNA synthesis, although the relationship between thyroid hormone receptors (TRs) and C9 requires further research. In fact, MC-LR caused immunotoxicity in fish and mammals by several toxicity mechanisms [20], and previous studies have demonstrated that MC-LR may affect the function of the complement system [108], regarded as an essential humoral system. 

The negative feedback regulatory response in fish, was also reported in juvenile zebrafish after acute MC-LR exposure (50–400 µg MC-LR/L) [100], in the same experimental conditions that the previous work of Gao et al. [103]. MC-LR exposure led to significant reductions of T4 and T3, and the toxin affected the transcription levels of genes involved in TH synthesis transport and metabolism, and the normal function of the thyroid. All the results confirmed that fish can trigger a compensatory mechanism to maintain TH homeostasis after continual exposure to the toxin.

Moreover, after parental exposure to MC-LR, thyroid disruption in F1 larvae zebrafish was demonstrated for the first time and highlighted the transgenerational toxicity of MC-LR [96]. The changes in the F1 offspring were decreased hatching and growth retardation, correlated with reduced THs levels. The decreased THs levels in the progeny may be due to abnormal gene transcription along the HPT axis in F1 larvae and thyroid dysfunction in adult female zebrafish, whereas the T4 and T3 levels were unchanged in males. The different expression pattern in both sexes of fish, could contribute to the sex related of THs levels, although further studies are needed to clarify the mechanisms. 

Recently, the effects of the coexistence of MC-LR and polystyrene nanoplastics (NSNPs) on the early growth of F1 zebrafish has been studied, and the combined exposure increased the parental transfer of MC-LR of the offspring and increased growth inhibition [102]. The decreased THs levels and the significant changes in the HPT axis gene expression indicated the thyroid disturbance caused by both contaminants could be the main cause of growth inhibition of F1 larvae. More studies are needed to explore the combination of cyanotoxins and other pollutants through the introduction of technologies, such as transcriptomics and proteomics [101].

Considering that Dios are key regulators of THs, and that the liver is the main organ that expresses the outer-ring deiodination (ORD) activity, the hepatic cell line of grass carp has been also chosen as an experimental model to investigate the effects of MC-LR on the activities of these enzymes [109]. Differences in the mRNA expression and activities of Dios were found after MC-LR exposure, decreasing Dio1 and Dio2 and increasing Dio3, and they could be responsible for the changes in THs levels and disturb the normal THs metabolic processes in fish.

Only one study investigated the thyroid endocrine disruption in developing zebrafish larvae exposed to MC-RR [94], and this MC-congener was able to change the transcription pattern of HPT-axis-related genes, except in the case of the thyroglobulin (TG) gene. Moreover, protein synthesis of TG was not affected, whereas NIS was significantly upregulated, in agreement with gene expression. In this study, the mRNA level of Dio1 decreased, but the transcription of Dio2 gene increased, in contrast to previous results (as mentioned above) obtained after MC-LR exposure by Yan et al. [99]: increased Dio1 and decreased Dio2 mRNA expressions. These facts could indicate that diverse MC-congeners exert thyroid toxicity by different mechanisms.

In addition, the thyroid toxicity of MCs in mammals has been poorly studied [3,97,98]. In the first work, in mice exposed to i.p. with MC-LR for 4 weeks, typical symptoms of hyperphagia, polydipsia, and weight loss with thyroid disfunction were found. Moreover, the animals showed glucose, triglyceride, and cholesterol metabolism disruptions. In this experiment, the high FT3 level found in exposed mice, together with a low FT4 level and a normal TSH concentration, was not consistent with the diagnosis of hyperthyroidism in the animals exposed to MC-LR. Increased expression of the Dio2 protein, increased expression of the THs receptor (TR α), and mTOR expression in the brain after exposure to the highest dose of 20 µg MC-LR/kg were reported. The increased plasma FT3 content regulating mTOR signaling was at first considered responsible for the increased food consumption and energy expenditure in the exposed mice. Furthermore, the glucose and lipid metabolic disorders during thyroid dysfunction could be due to the perturbed genetic expression of several genes of the key enzymes involved, which have been identified as being affected by thyroid hormone levels, after MC-LR exposure [97].

The chronic and low-dose effects of MC-LR on mouse thyroid tissues, exposed via the oral route, have also been investigated, as well as the toxin-induced apoptosis, lymphocyte infiltration, thyroid structural disorders, and changes in THs levels [98]. In this study, the expression of Dio3 increased in thyroid and peripheral tissues, and the phosphorylation level of the extracellular signal-regulated kinase (ERK), p38, and Mitogen-activated-protein kinase (MEK) was induced. This confirms that Dio3 and the p38/MAPK and MEK/ERK signaling pathways may play an important role in thyroid injury induced by MC-LR [110]. Moreover, the effects on thyroid hormones metabolism were also investigated in vitro by the same authors on thyroid follicular cells, Nthy-ori 3-1 cells. These cells accumulated MC-LR and, in agreement with the in vivo study, only the expression of Dio3 was increased, and activation of the p38/MAPK and MEK/ERK signal pathways was detected.

Finally, in an extensive study performed by Chen et al. [3] in female rats injected with a single dose of MC-LR (median lethal dose), several parameters were examined: histopathology of several organs, including thyroid tissue (thyroid follicular cells), concentrations of hormones in serum and gene expression of the HPA, HPG, and HPT axes. These authors suggested that MC-LR affected the HPA, HPG, and HPT axes. For the HPT axis, MC-LR induced higher TSH concentrations, although lower levels of TRH, FT3, and FT4 were reported. Globally, significant positive/negative correlations of concentrations of hormones were reported among the three axes, HPA, HPG, and HPT, and profiles of transcription of genes for synthesis of hormones and nuclear hormone receptors in thyroid, adrenal, and ovaries were altered. 

All these experiments open the door to new research by which to clarify the functional changes and mechanism of thyroid dysfunction in mammals (rats) after MC-LR exposure; to understand the potential risks of endocrine toxicity of cyanobacterial toxins, focused mainly on the mechanisms of effects of MC-LR on the different endocrine axes and hormonal functions. Thus, additional studies with mammalian models exposed through human relevant pathways would be welcome, as well as to investigate the effects of cyanobacterial blooms.

**Table 3:** Thyroid endocrine disruption of MCs.

Toxin	Experimental Model	Assays Performed	Concentration Ranges and Exposure Conditions	Main Results	References
Pure MC-LR standard	Zebrafish larvae	Extraction and measurement of thyroid hormones by ELISA. Expression in larvae of genes involved in HPT axis by RT-PCR: *CRH*, *TSH*, *NIS*, *TG*, *TRα*, *TRβ*, *Dio1*, and *Dio2.*	100, 300, and 500 µg/L for 96 hpf	Significant decrease in whole-body contents of T3 and T4. Significant up-regulation of *CRH*, *TSH*, *NIS*, *TG*, and *Dio1* genes. Transcription of TRs gene (*TRα*, *TRβ*) was downregulated in a concentration dependent manner, and *Dio2* gen was also downregulated.	[99]
Pure MC-LR standard	Zebrafish	Extraction and measurement of thyroid hormones by ELISA. Histology of thyroid follicles by hematoxylin and eosin. Expression of whole body *CRH*, *TSH*, *TPO*, and *TTR* genes by RT-PCR. Determination of deiodinase (Dio) activity.	1, 5, and 25 µg/L for 7, 14, 21, and 28 d	Significantly decreased contents of FT3 and T3 in whole body while T4 levels increased. Hypertrophy and hyperplasia of the thyroid follicle epithelial cells. Significant upregulation of genes involving TH synthesis. Reduced activities of both Dio1 and Dio2. Dio3 activity, however, was increased.	[95]
Pure MC-LR standard	*Gobiocypris rarus*	Extraction and measurement of thyroid hormones by radioimmunoassay. Histopathology. Expression of liver *NIS*, *TTR*, *TRα*, and *Dio (1,2,3*) genes by RT-PCR. Determination of Dio activity.	50, 100, and 500 µg/L for 7 d	T4 and T3 content significantly decreased. Transcription of the *NIS* gene was significantly downregulated with 500 µg/L MC-LR. Transcription of the *TTR* gene was significantly upregulated and downregulated in the 100 µg/L and 500 µg/L MC-LR exposure groups, respectively. The mRNA levels of *TRα* were also significantly downregulated in all the exposure groups. Similarly, *Dio2* and *Dio3* mRNA levels were significantly downregulated in all the exposure groups.	[107]
Pure MC-RR standard	Zebrafish larvae	Extraction and measurement of thyroid hormones by ELISA. Expression of different larvae genes by RT-PCR. Determination of TG and NIS levels by Western blot.	0.3, 1.0, and 3.0 mg/L for 96 hpf	T4 and T3 content significantly decreased. Transcription of *TSHβ*, *NIS*, and *Dio2* genes was upregulated, while *CRF, TRα,* and *Dio1* transcription was downregulated. TG protein was not affected. NIS protein expression was upregulated.	[94]
Pure MC-LR standard	Male and female Zebrafish	Extraction and measurement of thyroid hormones by ELISA. Histology of thyroid follicles by hematoxylin and eosin. Expression of *CRH* and *TSH* genes in brain, and *TTR*, *TRα*, and *TRβ* genes in liver by RT-PCR. Determination of Dio activity.	1, 5, and 25 µg/L for 7, 14, 21 and 28 d	Whole-body T3 and FT3 concentrations significantly decreased. No significant differences in T4 and FT4 levels. Thyroid follicles exhibited the same characteristics as the control group. Expression of *CRH* was downregulated, *TRα* expression was upregulated, and *TSH*, *TTR*, and *TRβ* expression was decreased followed by an increase at 21 and 28 days. Reduced activities of both Dio2 and Dio3. Dio1 activity decreased followed by an increase at 21 days.	[102]
Pure MC-LR standard	Male and female Zebrafish	Developmental toxicity in F1 generation. Extraction and measurement of thyroid hormones by ELISA. Expression of liver and brain genes involved in thyroid hormones metabolism by RT-PCR. Determination of TTR and TG levels by Western blot.	1, 5, and 25 µg/L for 45 d. After that, males and females were paired and allowed to spawn	T4 concentrations significantly decreased in F0 female. In F1 eggs, the whole-body T4 levels were significantly reduced. T4 levels in F1 larvae were reduced. In F1 larvae, only *TG* gene expression was upregulated, the rest of the genes (*CRH*, *TTR*, *Dio1*, *Dio2*, *TSH*, *TRα*) were downregulated. TTR protein expression was significantly downregulated, but TG protein expression was upregulated.	[96]
Pure MC-LR standard	Male and female Zebrafish	Extraction and measurement of thyroid hormones by ELISA. Determination of Dio activity. Expression in liver and brain of genes implicated in thyroid hormones metabolism by RT-PCR.	Adult zebrafish exposed to 50, 100, 200, and 400 µg/L for 24, 48, 72, and 96 h	T3 and T4 levels decreased. Expression levels of *TTR*, *TRα*, and *TRβ* significantly decreased. *CRH* and *TSH* expression levels initially decreased and then increased at 96 h. *TPO* expression levels showed different patterns of expression depending on the time of exposure and concentrations of MC-LR. Dio3 showed reduced activity, while Dio1 and Dio2 activities initially decreased followed by an increase at 72 and 96 h.	[103]
Pure MC-LR standard	Juvenile Zebrafish	Measurement of thyroid hormones by ELISA. Expression in whole zebrafish of genes involved in thyroid hormones metabolism by RT-PCR. Determination of Dio activity.	Juvenile zebrafish exposed to 50, 100, 200, and 400 µg/L for 24, 48, 72, and 96 h	T3 and T4 levels, decreased. The transcription levels of genes involved in TH synthesis, such as *CRH*, *TSH*, *TPO*, and *TTR*, were significantly decreased followed by an increase after MC-LR exposure. Transcription of the TH nuclear receptors (*tr-α* and *tr-β*) was significantly reduced during the exposure period. Dio3 showed a reduced activity, while Dio1 and Dio2 activities initially decreased followed by an increase at 72 and 96 h.	[100]
Pure MC-LR standard	Zebrafish larvae	Determination of MC-LR levels in larvae by ELISA. Measurement of T3 and T4 by ELISA. Expression in larvae genes implicated in the HPT axis (*NIS*, *CRH*, *TSH*, *TR*) and the GH/IGF axis (*gh, igf*).	F0 zebrafish exposed to 0.9, 4.5, and 22.5 µg/L of MC-LR, 100 µg/L of polystyrene nanoplastics (PSNPs) or their combination for 21 d	MC-LR and PSNPs significantly increased the accumulation of MC-LR in the F1 generation. MC-LR significantly reduced T4 levels in F1 larvae. After co-exposure, the concentration of T4 decreased further. T3 level of F1 larvae was also significantly reduced in the high-concentration MC-LR group. T3 content of larvae significantly decreased in all PSNPs + MCLR groups. Parental exposure to PSNPs and MC-LR had more pronounced effects on thyroid hormone levels in larvae. When exposed to 4.5 μg/L MC-LR treatment group, the transcription of *tr-α*, *tr-β*, and *Dio2* were significantly downregulated, and the mRNA levels of *TG, TTR*, and *Dio1* were significantly increased. When parents were exposed to 22.5 μg/L MC-LR, the mRNA levels of *CRH*, *tr-α*, *tr-β*, and *Dio2* were significantly decreased, and the transcriptions of *TG, TTR,* and *Dio1* were significantly multiplied in F1 larvae.	[101]
Lyophilized biomass of *Microcystis aeruginosa*	*Xenopus laevis* tadpoles	Determination of corticosterone and aldosterone. mRNA expression of hormones concerning development of the brain and expression of genes associated with stress, biotransformation, and oxidative stress in the liver by qPCR.	10 and 50% lyophilized biomass of *Microcystis aeruginosa* for 7 or 21 d	No changes in *TSHβ* and *FSHβ* expression, while *LHβ* expression significantly increased in 10% group after 21 days. Both aldosterone and corticosterone levels increased at 21 days in 50% group compared to the control. Expression of genes associated with biotransformation and oxidative stress revealed no significant differences.	[104]
Pure MC-LR standard	L8824 cell line	Cell viability by MTT test. Expression of Dio genes by RT-PCR. Determination of Dio activity.	1, 10, 100, and 1000 µg/L for 24 and 48 h	Cell viability was not significantly affected. Expression of *Dio1* and *Dio2* genes was downregulated, *Dio3* expression was upregulated. Decreased activities of both Dio1 and Dio2, whereas Dio3 activity was increased.	[109]
Pure MC-LR standard	Male Balb/c mice	Determination of thyroid hormones by ELISA. Quantification of CHOL, HDL, LDL, and TG plasma content by colorimetric methods. Expression of Dio2, TRα, and mTOR in brain by Western blot.	5 or 20 µg/Kg b.w. i.p. injection for 4 weeks	Blood biochemistry showed decreased levels of FT4, GLU, TG, CHOL, and HDL, however, FT3 and LDL levels significantly increased. Brain protein levels of mTOR, Dio2, and TRα significantly increased.	[97]
Pure MC-LR standard	Female BALB/C mice	Histology of thyroid. Determination of apoptosis levels in testes by TUNNEL staining. Quantification of FT3, FT4, and TSH levels. Expression in thyroid of Dio genes by RT-PCR. Protein levels of ERK, p38, and MAPK by Western blot.	1, 10, 20, and 40 µg/L for 6 months, oral administration	No significant collapsed areas in the follicular thyroid carcinoma. Increase in the number of apoptotic cells at 40 μg/L MC-LR. FT3 and FT4 were significantly decreased at 40 μg/L. TSH was significantly upregulated at 20 μg/L and 40 μg/L. *Dio3* was significantly upregulated in skin and thyroid at 40 μg/L. MC-LR at 10, 20, and 40 μg/L induced phosphorylation of ERK, p38, and MAPK.	[98]
	Nthy-ori 3-1 cell line (thyroid follicular epithelial cells)	CCK-8 assay. FDA and PI staining for morphologic evaluation. Flow cytometry for cell apoptosis measurement. Expression of Dio genes by RT-PCR.	0.5, 5, 50, and 500 nM for 12–24 h	MC-LR accumulated in the cells treated with 50 and 500 nM. Cell viability significantly decreased. Expression of Dio3 was increased following exposure to 5, 50, and 500 nM MC-LR. MC-LR exposure at 5, 50, and 500 nM induced phosphorylation of ERK, p38, and MAPK protein expression in a dose-dependent manner. Inhibition of both signal pathways (ERK and MAPK) could significantly reverse the upregulated *Dio3* expression induced by MC-LR.	
Pure MC-LR standard	Female SD rats	Histopathology observation of several organs, including thyroid gland. Quantification of serum levels of CRH, ACTH, CORT, GnRH, T, E2, TRH, FT4, FT3, LH, FSH, and TSH by ELISA. Hypothalamus, pituitary and thyroid mRNA expression of *crh, gr, gnrh1, erα, erβ, fshr, lhr, 3βhsd,* and *17βhsd* by qRT-PCR.	i.p. injections of 36.50, 54.75, or 73.00 μg MC-LR/kg b.w for 24 h	In ovaries, rats exposed to 54.75 or 73 μg MC-LR/kg exhibited obvious hyperemia, cytoplasmic loss, abnormal nuclear change, and nuclear dissolution. Necrosis of local granular cells in rats exposed to 73 μg MC-LR/kg. Broken nuclei, necrosis of follicular epithelial cells, and reduced intracellular colloid in thyroid gland. Serum concentrations of CRH, ACTH, and CORT significantly decreased. Lower concentrations of GnRH and E2, but higher concentrations of LH, FSH, and T. Concentrations of TRH, FT4, and FT3 significantly decreased in treated rats, but high TSH concentration was recorded. Expressions of genes among HPA, HPG, and HPT axes were diverse. In the hypothalamus, transcripts of *thrβ* were lower in rats exposed to 73 μg MC-LR/kg, while there were no significant changes in expression of *trh, tshr,* or *thrα*. In the pituitary, there were no significant effects on mRNA expressions of *trhr*, *tshβ*, *thrα,* or *thrβ*. In the thyroid, there were no significant alterations in abundances of transcripts of *tshr*, *thrβ*, *tg*, or *tpo*. When exposed to 36.5 μg MC-LR/kg bm, expression of *ttf1* was significantly upregulated. Treatment with 54.75 or 73 μg MC-LR/kg bm caused significant upregulation of diol2 and diol3.	[3]

ACTH: corticotropin; 3βhsd: 3beta-hydroxysteroid dehydrogenase; CHOL: cholesterol; CRH: corticotropine-releasing hormone; CORT: cortiscosterone; CRF: corticotropin-releasing factor; Dio (1,2,3): iodothyronine deiodinase; ERK: extracellular signal-regulated kinase; FDA: fluorescein diacetate; fshr: follicle stimulating hormone receptor; FT3: free triiodothyronine; FT4: free thyroxin; GH/IGF: growth hormone/insulin-like growth factors; GLU: glucose; gr: glucocorticoid receptor; GnRH: gonadotropin-releasing hormone; HDL: high density lipoproteins; HPT: hypothalamic-pituitary-thyroid axis; i.p.: intraperitoneal; LDL: low density lipoproteins; lhr: luteinizing hormone receptor; MAPK: mitogen-activated protein kinases; mTOR: mammalian target of rapamycin; MTT: 3-(4;5-dimethylthiazol-2-yl)-2;5-diphenyltetrazolium bromide; NIS: sodium/iodide symporter; PI: propidium iodide; PNPs: polystyrene nanoplastics; RT-PCR: real time polymerase chain reaction; T3: triiodothyronine; T4: thyroxin; TG: thyroglobulin; TH: thyroid hormones; TPO: thyroperoxidase; TR (α, β): thyroid hormone receptor; TSH: thyroid-stimulating hormone; TTR: transthyretin.

## 3. Cylindrospermopsin

Studies focused on the potential endocrine-disrupting effects of CYN are very scarce (Table 4). Some estrogenic effects have been detected with cyanobacterial extracts containing CYN [49], but information on the estrogenic potency of CYN is very limited [50]. 

Regarding the scientific literature, the first study in which pure CYN exhibited this activity was reported in primary human-IVF-derived granulosa cells exposed to low concentrations (0–1 µg CYN/mL) for 2–6 h or for 24–72 h. In this model, the human chorionic gonadotropin (hCG)–stimulated progesterone production was inhibited when exposed to a non-cytotoxic concentration of CYN (1 µg/mL) for 24 h [111]. They also found that although CYN was not cytotoxic and it did not affect hCG-stimulated estrogen production after 6 h, the toxin did abolish hCG-stimulated progesterone production. However, in a second experiment, using IVF-derived cells obtained selectively from women with normal fertility status, CYN up to 3 μM (1.25 µg/mL for 6 h) was not cytotoxic, and did not alter progesterone or estrogen production, with or without hCG stimulation. Protein synthesis was significantly inhibited by 3 μM CYN alone, and CYN in the presence of hCG caused a concentration-dependent decrease in global protein synthesis, but had no effect on the synthesis of steroid hormones. This suggests that StAR and CYP450 aromatase (CYP450arom) protein synthesis was not inhibited by CYN [112].

Some aqueous extracts of cyanobacteria, such as *Aphanizomenon flos-aquae* (a CYN productor), have been shown to be active in the human breast carcinoma cell line MVLN [49]. The only extract that showed clear dose-dependent antiestrogenic activity in co-exposure with E2 was *Aph. flos-aquae* PCC, containing 3100 µg CYN/g d.w. Moreover, the potency was not correlated with CYN concentrations, suggesting that the activity was also due to other compounds. 

Liu et al. [113] investigated the estrogenic activity of CYN (2.4 × 10^−7^ M to 2.4 × 10^−12^ M) by a yeast estrogen screen (YES) assay and after oxidation of the toxin. CYN was anagonist in the YES assay, and its binding affinity to the estrogen receptor was linked to its intrinsic properties. The toxin modulated E2 estrogenic activity, resulting in non-monotonic responses, and this behavior is common to xenobiotics, known as estrogen-actived chemicals (EACs). After treatment with oxidative compounds, the by-products obtained had reduced binding affinity to estrogen receptors in comparison to the parent toxin [113]. 

Recently, the direct role of CYN on testicular function and spermatogenesis in aquatic organisms has been studied using ex vivo cultures of zebrafish testes as experimental models [114]. After exposure to CYN (250–1000 µg/L CYN for 24 h and 7d), the toxin reduced the basal- and gonadotropin-induced process of spermatogenesis, and it may have contributed to a decrease in fertility. Moreover, CYN inhibited all stages of spermatogenesis in zebrafish testes; this disruption of the spermatogenesis process could indicate that CYN can impair male reproduction. Significant changes in fshr, lhr, and insulin-like grown factor 3 (*igf*) transcript levels were also found, and T secretion was increased as well. These findings contributed to a better understanding of the mechanisms involved on the effects of CYN on male reproduction by inducing apoptosis and altering gonadotropins, as well as changing production of T and *igf3*. The last fact, inhibition of *igf3* by CYN, could be a contributing factor in the mechanisms of CYN-induced impairment of differentiated spermatogonia development [114].

Thus, considering the relevance of CYN, knowledge of its potential endocrine disruption properties should be further investigated. Reports so far, although scarce, have suggested that CYN is a potent EDs on different models, as well as CYN-containing extracts.

**Table 4:** CYN estrogenic/antiestrogenic and androgenic/antiandrogenic effects and thyroid endocrine disruption.

Toxin	Experimental Model	Assays Performed	Concentration Ranges and Exposure Conditions	Main Results	References
CYN extract from *Cylindrospermopsis raciborskii*	Male and female C57/BL6 mice	Estrous cycle evaluation. Sperm counting. P, E2, and T determination by RIA. Gene expression in pituitary and testes.	20 or 64 µg CYN/Kg body weight.	A single injection of 64 µg CYN/kg b.w. in females induced an impairment in the estrous cycle and a decrease in P levels. In males, weekly doses of 20 µg CYN/kg b.w increased T levels and spermatozoa cells.	[115]
Pure CYN standard	Zebrafish embryos	Morphometric analysis. Expression of genes involved in oxidative stress and thyroid hormones function by RT-qPCR.	10, 50, 100, 500, 1000, and 2000 µg/L for 120 hpf	Decreased growth and increased developmental abnormalities. CYN induced changes on thyroid-hormone-related genes (*tr1a*, *Dio1*, *Dio3*), which could be involved in the induction of thyroid disruption.	[116]
Pure CYN standard	Adult male zebrafish	Histological analysis of testes. Determination of testosterone by ELISA. Caspase-3 activity assay. Gene expression (*lhr*, *fshr*, *igf3*, etc.) by RT-qPCR.	250, 500, or 1000 µg/L for 24 h or 7 d.	CYN inhibited all stages of spermatogenesis at the level of testis. Significant changes in *fshr*, *lhr*, and *igf3* transcript levels were found, and T secretion was increased.	[114]
Lyophilized CYN	Primary human IVF-derived granulosa cells	MTT cytotoxicity. Estrogen and P measurement by RIA.	0–1 µg/mL of CYN for 2, 4, or 6 h and 24, 48, or 72 h.	hCG–stimulated P production was inhibited at 24 h. CYN was not cytotoxic, and it did not affect hCG-stimulated estrogen production after 6 h. The toxin did abolish hCG-stimulated P production.	[111]
CYN extracted from *Cylindrospermopsis* *raciborskii*	IVF-derived cells, selectively obtained from women with normal fertility status	Estradiol and P measurement by RIA.	0.1, 0.3, 1, and 3 µM for 6 h	CYN up to 3 μM was not cytotoxic and did not alter production of P or estrogen, with or without hCG stimulation. At 3 µM CYN, protein synthesis was inhibited. CYN in the presence of hCG caused a concentration-dependent decrease in global protein synthesis.	[112]
Aqueous extracts of CYN-producer cyanobacteria	Human breast carcinoma cell line MVLN	Determination of estrogen-receptor-mediated effects.	0.001–0.25 g dw/L for 24 h	*Aph. flos-aquae* showed concentration-dependent antiestrogenic activity in co-exposure with E2.	[49]
Pure CYN standard	Genetically modified strains of *Saccharomyces cerevisiae*	Estrogenic activity by YES.	2.4 × 10^−7^–2.4 × 10^−12^ M for 24 h	CYN modulated the E2 activity, resulting in non-monotonic responses. After treatment with oxidative compounds, the byproducts obtained reduced binding affinity to estrogen receptors in comparison to the parent toxin.	[113]

E2: estradiol; hGC: human corionic gonadotropin; hpf: hours post fertilization; MTT: 3-(4, 5-dimethylthiazolyl-2)-2,5-diphenyltetrazolium bromide); P: progesterone; RIA: radioimmunoassay; T: testosterone; YES: yeast estrogen screen.

The same authors investigated the adverse effects of CYN (0–2000 µg/L CYN) on zebrafish embryonic development [116], and numerous changes were observed: decreased growth, increased developmental abnormalities, such as pericardial and yolk sac edema, as well as swim bladder absence. In addition, CYN induced changes in thyroid-hormone-related genes (*tr1a*, *Dio1*, *Dio3*) which could be involved in the induction of thyroid disruption. Globally, the authors indicated that CYN exposure alters functions related to thyroid hormones, oxidative stress, and osmoregulation, all of them key components for normal embryo development [116]. These results confirmed the adverse effects on embryonic development induced by CYN in zebrafish exposed to the toxin (2–2000 nM) previously reported by Wang et al. [117], who indicated that they may be associated with oxidative stress and apoptosis. 

Finally, the disruption of the estrous cycle and its effects on spermatogenesis in vivo in female and male mice was recently investigated [115]. A single i.p. injection of 64 µg CYN/kg b.w. in females induced an impairment in the estrous cycle and a decrease in progesterone levels. In males, weekly i.p. doses of 20 µg CYN/kg b.w given to several groups (1, 2 or 4 doses) increased T levels in groups administered with 1–2 doses and induced increases in spermatozoa cells. All of these results indicate that CYN interferes in the mammalian reproductive system, and may cause infertility, although further studies should be carried out to confirm this dysfunction in mammals and the mechanisms involved.

## 4. General Discussion and Final Remarks

Endocrine disruption effects have gained relevance from a toxicological point of view due to the wide number of xenobiotics exerting disruptive properties and their contribution to different illnesses and health effects, in both humans and the environment. Moreover, very different mechanisms can be involved (i.e., interaction with receptors, changes in metabolism of endogenous hormones, interference with feedback regulation and neuroendocrine cells, genomic instability by interference with the spindle figure can play a role, etc.), as reported by De Coster and van Larebeke [9].

MCs and CYN are cyanobacterial toxins that, although targeting the liver, have been shown to also induce endocrine disruption effects, as evidenced in the present review manuscript.

The endocrine disruption activity of MCs has been more extensively investigated in comparison to CYN. Moreover, some of the studies have been performed with cyanobacterial cultures that contain other kinds of compounds that could be responsible of the effects observed (i.e., [49,65,78,104], etc.). If we focus on pure toxins, the available results are highly variable. Thus, Hou et al. [48] reported that MC-LR is unlikely to cause estrogenic responses via binding ER, as its chemical structure is different to other known EDCs, but there are many studies that have evidenced MCs ED effects by other pathways. These include, among others, pathological damage in related organs and cells such as the testis (i.e., [61]), ovarian cells (i.e., [44,66]), Leydig cells (i.e., [83]), or GnRH neurons [46]. Moreover, these cells can suffer from apoptosis and toxic effects mediated by ROS [84] or immune cells [73]. Additional effects/mechanisms include: decreased gonad-somatic index [41], changes in transcriptional responses of HPG-axis related genes [43,47,60], HPI-axis-related genes [93], HPT-axis-related genes [99], steroidogenesis-related genes [76]; disruption of the GH/IGFs system [59]; activation of the ERK1/2 signaling pathway [64], changes in the activity of GnRH transcription factors [74], interference with steroidogenic enzymes [91], changes in hormone levels (i.e., [66,84,96]), etc.

Conversely, pure CYN did show binding affinity to the estrogen receptor [113]. Although studies dealing with CYN are very limited, and the results obtained were also variable, it showed ED effects mediated by changes in the transcript levels of related hormones, apoptosis induction [114], changes in thyroid-hormone-related genes [116], oxidative stress [117], or alterations in hormone levels [114,115].

In any case, none of the studies reviewed, for either MCs or CYN, were performed following official OCDE guidelines, and the experimental designs and exposure scenarios have been very different. This makes it difficult to establish certainties regarding the ED activity of cyanotoxins. Moreover, there was a study conducted with MC-LR [3] and a study conducted on CYN [114] that reported non-monotonic responses, a phenomenon frequently described for endocrine disruptors, but without standardized approaches in a risk assessment context [118]. 

Therefore, and based on the scientific literature reviewed, the cyanotoxins MCs and CYN are potential endocrine disruptors, but further research is required, particularly for CYN, but also for MCs, considering: the use of OCDE guidelines; the use of advanced technologies such as transcriptomics and proteomics; combined exposures of cyanotoxins with other pollutants; and potential non-monotonic dose-response relationships.

## Data Availability

Not applicable.
